# Parameter estimation of proton exchange membrane fuel cells using enhanced Fourier transform optimizer with adaptive learning mechanisms

**DOI:** 10.1038/s41598-026-60492-z

**Published:** 2026-07-08

**Authors:** Ramakrishna Raghutu, Tummala S. L. V. Ayyarao, G. Sasikumar, U. Siddaraj

**Affiliations:** 1 Department of Electrical and Electronics Engineering, GMR Institute of Technology (GMRIT) (Deemed to be University), Rajam, Andhra Pradesh 532127 India; 2 VNR Vignana Jyothi Institute of Engineering and Technology, Hyderabad, India; 3 Manipal Institute of Technology, Manipal Academy of Higher Education, Manipal, India

**Keywords:** Proton exchange membrane fuel cell, Enhanced Fourier transform optimizer, Parameters estimation, Metaheuristic optimization, Fourier transform optimizer, Energy science and technology, Engineering, Mathematics and computing

## Abstract

The seven unknown parameters in the Amphlett semi-empirical proton exchange membrane fuel cell (PEMFC) model are critical to accurate performance prediction, system design, and degradation diagnosis. Gradient-based methods are often unreliable in this context because the sum-of-squared-errors (SSE) objective is highly nonlinear and multimodal, while existing population-based approaches tend to exhibit substantial run-to-run variability. To address these challenges, this paper proposes the Enhanced Fourier Transform Optimizer (EFTO), a hybrid adaptive algorithm that combines a success-history differential evolution branch with elite-guided mutation and a covariance-adaptive sampling branch, dynamically balanced through an online branch-selection mechanism. On the IEEE CEC 2017 benchmark suite (28 functions, D = 50, 30 independent runs), EFTO achieved a Friedman rank of one with an average rank of 1.607 and a Wilcoxon *p* ≤ 1.17 × 10⁻^3^ against six competing algorithms. A supplementary comparison with three recognized state-of-the-art solvers found no statistically significant performance differences relative to two of the three solvers (*p* > 0.05), while EFTO recorded the lowest average runtime of 8.653 s. When applied to three commercially validated PEMFC stacks, EFTO achieved machine-precision reproducibility, with a standard deviation below 10⁻^14^ V^2^ across 30 independent runs and best SSE values of 2.0656 V^2^ (NedStack PS6), 1.0564 V^2^ (AVISTA SR-12), and 0.8139 V^2^ (Ballard Mark V). These results were statistically confirmed by Wilcoxon signed-rank tests (R⁺ = 465, *p* = 1.73 × 10⁻⁶). Component wise ablation showed that the covariance-adaptive sampling mechanism was the most influential component, with removal of the covariance-adaptive sampling mechanism causing a 98.7% degradation in SSE (*p* = 1.09 × 10⁻^24^). Stack-specific sensitivity analysis further demonstrated that the identified parameters remain physically consistent with the electrochemical operating regime of each stack, establishing EFTO as a competitive and consistent tool for PEMFC parameters identification on the evaluated benchmark configurations.

**Supplementary Information:**

The online version contains supplementary material available at 10.1038/s41598-026-60492-z.

## Introduction

The global need to decarbonize the energy sector has driven increased research activity surrounding hydrogen-fueled proton exchange membrane fuel cells (PEMFCs) as a premier clean energy technology. PEMFCs directly transform the chemical energy of hydrogen into electricity without combustion, offering high energy conversion efficiency, no local pollutant emissions, rapid startup capability, and an operating temperature of 60–100 °C^1–3^. The high efficiency and zero-emission characteristics of PEMFCs have led to the widespread deployment of PEMFCs in automotive powertrains^4^, stationary micro-CHP systems^5^, and distributed backup power infrastructure^6^. Accurate mathematical modelling of PEMFC electrochemical behavior under varying operating conditions is therefore essential for system design, performance optimization, fault diagnosis, and control strategy development^7,8^. The semi-empirical model suggested in^9^ is the most widely adopted framework for PEMFC polarization characteristics. Using seven unknown parameters [ξ₁, ξ₂, ξ₃, ξ₄, λ, β, R_c_] that must be extracted from experimental current–voltage (I-V) data^9,10^, the model quantifies three dominant sources of voltage loss: activation overpotential, ohmic resistance, and concentration polarization. Parameters estimation is intrinsically nontrivial, because the SSE objective function is highly nonlinear and multimodal, and strict physical constraints limit the feasible region to a small fraction of the seven-dimensional search domain^11^. Classical gradient-based deterministic methods are sensitive to initial conditions, often converging prematurely at local minima, and do not maintain sufficient stochastic diversity for traversing this complicated landscape^12^. Due to the derivative-free stochastic search mechanism of nature-inspired metaheuristic algorithms, nature-inspired metaheuristic algorithms have emerged as the methods of choice for PEMFC parameters estimation^13–16^.

Parameters estimation is an area that has seen tremendous development over two decades, from early population-based approaches to increasingly sophisticated adaptive techniques summarized in Table 1. A hybrid genetic algorithm combined with the Nelder-Mead simplex method was among the earliest hybridization strategies proposed for PEMFC parameters estimation^17^, while differential evolution was shown to outperform gradient-based methods on PEMFC models^18^. An adaptive inertia-weight particle swarm optimizer was subsequently introduced^19^, and the Bird Mating Optimizer was validated on the Ballard Mark V stack^20^. The swarm intelligence era brought further advances, including the Grey Wolf Optimizer applied across multiple stacks^13^, a hybrid GWO-DE variant^21^, and LSHADE-EpSin with sinusoidal adaptation of control parameters achieving consistently low SSE across multiple stacks^22^. Further contributions improved solution quality with an enhanced Archimedes Optimization Algorithm^23^, Harris Hawks Optimization^14^, Modified Golden Jackal Optimizer combined with particle swarm^24^, and a blended artificial bee colony-DE framework^25^. War Strategy Optimization^16^ and the Youngs Double-Slit Experiment Optimizer^26^ demonstrated comparable accuracy on the NedStack PS6, Ballard Mark V, and AVISTA SR-12 benchmarks. Most recently, the Fourier Transform Optimizer (FTO) was introduced in^27^, where population evolution occurs in the frequency domain through the Discrete Fourier Transform, and as such has a physically inspired search mechanism that cannot be reduced to spatial operations.

**Table 1:** Summary of representative metaheuristic algorithms applied to PEMFC parameters estimation.

Reference	Algorithm	Application	Key contribution
^18^	Differential Evolution	N/A	DE outperforms gradient-based methods; first population-based PEMFC study
^12^	Gradient-Based Optimizer	250W, SR-12, BCS	Physics-informed deterministic strategy for PEMFC parameters extraction
^20^	Bird Mating Optimizer	Ballard Mark V	BMA estimates seven PEMFC parameters with competitive accuracy
^13^	Grey Wolf Optimizer (GWO)	Ballard, BCS, SR-12	GWO applied across multiple PEMFC stacks; key benchmark study
^21^	Hybrid GWO-DE	250W, AVISTA, Ballard, BCS	DE mutation and crossover operators enhance original GWO convergence
^22^	LSHADE-EpSin	250W, BCS, SR-12, NedStack	Sinusoidal adaptation integrated into LSHADE; low SSE across multiple stacks
^23^	Improved AOA	NedStack PS6, Nexa	Improved Archimedes Optimization achieves accurate parameters identification
^14^	Harris Hawks Opt. (HHO)	250W, SR-12, BCS	Two-phase HHO evaluated across multiple PEMFC configurations
^24^	Modified Golden Jackal	NedStack, BCS 500W	Golden Jackal hybridised with PSO for improved extraction accuracy
^25^	Hybrid ABC-DE	Multiple stacks	Artificial bee colony combined with DE for efficient PEM modelling
^16^	War Strategy Opt. (WSO)	NedStack, Ballard, AVISTA	WSO achieves competitive SSE on three PEMFC benchmark stacks
^26^	YDEO	Horizon, BCS, NedStack, 250W	Physics-inspired YDEO achieves low SSE across four PEMFC configurations
^27^	Fourier Transform Opt. (FTO)	NedStack, Ballard, BCS	Frequency-domain recombination; novel search paradigm for PEMFC
^28^	L-SHADE	CEC 2014 benchmarks	Linear population size reduction with success-history parameter adaptation
^19^	Adaptive PSO	N/A	Adaptive inertia-weight mechanism improves PSO convergence on PEMFC
^17^	GA-Nelder-Mead	250W	Hybrid GA with Nelder-Mead simplex; pioneered hybridisation for PEMFC optimization
^29^	jSO	CEC 2017 benchmarks	Progress-aware F/CR scaling with success-history parameter memory
^30^	Adaptive covariance search	Continuous global optimization	Covariance matrix adaptation with cumulative step-size control
^31^	Secant Optimization Algorithm	Global optimization benchmarks	Numerical secant method principles for efficient global convergence
^32^	Schrödinger Optimizer	Engineering optimization	Quantum wave-particle duality for exploration–exploitation balance
^33^	Improved Moth Flame with Local Escape	Photovoltaic parameters estimation	Local escape operators enhancing solution quality in PV identification
^34^	Quadratic Interpolation PSO	Solar PV parameters estimation	Quadratic interpolation with local search for PV parameters accuracy
^35^	Quadratic Interpolation Salp Swarm	Large-scale global optimization	Local escape operator for scalable high-dimensional optimization
^36^	SSALEO	Large-scale constrained optimization	Scalable framework for real-world constrained benchmark problems
^37^	Modified Gradient Search	Solar PV parameters extraction	Quasi-Newton hybrid with gradient-based local refinement
^38^	KRMG (KOA + RPO + MO + GWO)	CEC2014/2019/2022; constrained engineering design	Four-component hybrid with proportional population shrinkage and staged mutation tuning
^39^	INFONM (INFO + Nelder-Mead)	PEMFC 7-parameters; NedStack PS6, BCS 500 W, 250 W, Ballard Mark V	INFO for global search; Nelder-Mead for local refinement; SSE-based objective
^40^	25 MH algorithms (comparative)	PEMFC identification; six stacks	Large-scale SSE benchmark; algorithms ranked via convergence, boxplot, radar, and correlation analyses
^41^	SaDE + *g*-function model	PEMFC; Ballard Mark V, BCS 500 W, NedStack PS6	Numerically stable *g*-function replaces Lambert W; self-tuning SaDE; RMSE reduced up to 6.65%
^42^	Ali-Baba and Forty Thieves (ABFT)	PEMFC 7-parameters; five stacks	Constrained SSE minimisation; foraging–looting phases balance exploration–exploitation
^43^	Enhanced Meta-Evolutionary DE (EMEDEA)	PEMFC; three stacks	Adaptive mutation factor and crossover rate; SSE-driven; outperforms recent benchmarks

The rationale for the algorithmic strategies in EFTO rests on solid theoretical foundations from the metaheuristic literature^28–30^, providing assurance that each design decision is based on credible prior work and making the novel synthesis of these strategies arguably the key contribution of this new framework [^63–65^ ]. A recent extensive benchmarking of 25 metaheuristic algorithms on six PEMFC stacks^40^ has proved that robust parameters identification is still not solved. Complementary PEMFC-specific advances, including hybrid local-search extraction^39^, g-function modelling with self-adaptive DE^41^, constrained SSE-based identification^42^, and adaptive mutation DE frameworks^43^, further confirm this open challenge. At the same time, recent developments such as local escape operator variants for photovoltaic parameters estimation^33,34,37^, large-scale constrained optimization frameworks^35,36,38^ and quantum-inspired and secant-based metaheuristics^31,32^ show a more general shift toward adaptive hybrid strategies that inspires the present work.

Although the approaches documented in Table 1 have made substantial progress, the approaches suffer from four remaining structural limitations that motivate the present work. First, purely random initialization gives no systematic coverage guarantee in the PEMFC parameter space and hence solution quality is sensitive to random seed selection. Second, algorithmic control parameters such as scaling factor and crossover probability are mostly either fixed or schedule-based rather than explicitly adapted from the historical success of different search configurations, making the parameters less reactive to the evolving landscape [^66,68,69^ ]. Third, most algorithms have no principled method with which to recover from stagnating individuals; stagnating individuals simply recover by random re-initialization without directional corrective strategies [^70,71^ ]. Fourth, the exploration versus exploitation tradeoff is not continuously and mathematically principled across the lifetime of a search [^72–75^ ].

These limitations are evident in the large standard deviations and worst-case SSE reported for WOA^44,45^, AOA^23^ and GWO^13^ over independent runs, which further establishes that run-to-run reproducibility is still a problem. The No Free Lunch theorem^46^ rigorously states that no one algorithm can outperform all others all the time, providing a continuing scientific basis for developing methodologically grounded adaptive frameworks.

The FTO^27^ was adopted as the algorithmic foundation because the frequency-domain search strategy of FTO facilitates simultaneous exploration of different spatial scales. Low-frequency components describe broad structural patterns, whereas high-frequency components represent finer details, resulting in a search behavior that is geometrically distinct from that of traditional spatial operators. The DFT representation is also naturally a full coupling of all the variables at once, which corresponds precisely to tackling the non-separability of the PEMFC SSE landscape. 

However, the FTO also has five structural deficiencies. In the first place, both fixed scaling factor F = 0.5 and Lévy exponent β_L_ = 1.5 inhibit adaptive landscape response. Second, since in the frequency-component schedule reaches zero at iteration 858 for D = 7 (the last 14.2% of the run), the schedule deactivates the primary operator for that duration. Thirdly, random initialization does not systematically cover the parameter space that is constrained. Fourth, there are no corrective mechanisms for stagnating individuals. Fifth, hard boundary clipping leads to spectral discontinuities that corrupt DFT-based perturbations. This targeted boosting, in turn, inspires the development of EFTO since each of these deficiencies can be specifically and independently addressed.

To overcome these deficiencies, the EFTO utilizes eight synergistic mechanisms: opposition-based learning initialization (M1), success-history adaptive parameter memory (M2), elite-guided differential mutation (M3), covariance-adaptive population sampling (M4), quasi-oppositional stagnation recovery (M5), midpoint boundary handling (M6), boundary exploration probe (M7) and terminal-phase coordinate local search (M8). One or more of the identified FTO deficiencies has a direct resolution through each mechanism, as formally established through the component wise ablation study in Section "Results and discussion".

## Research objectives

The specific objectives of this study are:

i. To identify and address the structural limitations of the baseline Fourier Transform Optimizer that impede reliable convergence on nonlinear multimodal landscapes;
ii. To develop a hybrid adaptive metaheuristic that integrates success-history differential evolution with covariance-adaptive population sampling, regulated by an online branch-selection strategy, to improve solution consistency and convergence reliability;
iii. To validate the proposed algorithm on the IEEE CEC 2017 benchmark suite against both general-purpose and state-of-the-art metaheuristic algorithms using standardized statistical evaluation;
iv. To apply and evaluate the proposed algorithm for PEMFC parameters identification across three commercially validated stacks and assess the physical consistency of identified parameters through sensitivity analysis; and
v. To conduct component wise ablation to quantify the individual contribution of each enhancement mechanism and establish the necessity of the proposed architecture.

### Research contributions and novelty

#### Core research contributions

i. A novel EFTO algorithm is proposed that addresses five structural deficiencies of the baseline FTO through eight synergistic mechanisms. Component wise ablation confirms co-designed synergy: disabling all mechanisms causes + 3032.8% SSE degradation (*p* = 2.30 × 10⁻^2^⁶) and CAPS removal alone causes + 98.7% (*p* = 1.09 × 10⁻^24^).
ii. On the IEEE CEC 2017 suite (28 functions, D = 50, 30 runs), EFTO achieves Friedman rank 1 (average rank 1.607, *p* ≤ 1.17 × 10⁻^3^ against six algorithms) with the lowest runtime of 8.653 s among all state-of-the-art competitors.
iii. For PEMFC parameters identification, EFTO achieves machine-precision reproducibility (Std < 10⁻^14^ V^2^) on all three stacks, with best SSE of 2.0656 V^2^ (NedStack PS6), 1.0564 V^2^ (AVISTA SR-12), and 0.8139 V^2^ (Ballard Mark V), statistically confirmed (R⁺ = 465, *p* = 1.73 × 10⁻⁶).
iv. Temperature and pressure sensitivity analyses at three operating levels per stack physically validate the EFTO-identified parameters across a realistic operating envelope, confirming physical consistency beyond curve-fitting accuracy.
v. Parameter sensitivity was examined using a one-at-a-time ± 5% variation approach. The analysis showed that ξ₁ and ξ₂ exert the greatest influence on the model response for all three stacks, indicating the importance of ξ₁ and ξ₂ for both model reduction and engineering design considerations.

### Novelty of the proposed EFTO

i. EFTO is the first variant within the FTO family to incorporate covariance matrix adaptation in place of frequency-domain recombination, enabling effective handling of rotated, ill-conditioned, and non-separable optimization problems.
ii. The dimension-aware heuristic switching strategy automatically deactivates low-dimensional operators at high dimensions, ensuring computational efficiency without manual reconfiguration.
iii. The adaptive branch probability mechanism leverages online success rates to dynamically adjust the balance between the differential evolution and covariance-adaptive search branches, a meta-learning strategy empirically validated through component-wise ablation (Section "Results and discussion").

The remainder of this paper is organized as follows. Section "PEMFC mathematical model" presents the PEMFC mathematical model and the parameters estimation problem formulation. Section "Proposed enhanced Fourier transform optimizer (EFTO)" describes the proposed EFTO algorithm, including the complete mathematical derivation of all eight-enhancement mechanisms. Section "Results and discussion" reports the CEC 2017 benchmark validation results and the PEMFC parameters estimation results across all three benchmark stacks. Section "Conclusion" draws the conclusions.

## PEMFC mathematical model

A PEMFC stack comprising ${\mathrm{N}}_{{{\mathrm{cells}}}} { }$ series-connected cells converts the chemical energy of hydrogen and oxygen into electrical energy through electrochemical reactions at the anode and cathode. The governing half-reactions are^9,47^:

1 $${\mathrm{Anode}}:\;2{\mathrm{H}}_{2} \to 4{\mathrm{e}}^{ - } + 4{\mathrm{H}}^{ + } ; {\mathrm{Cathode}}: 4{\mathrm{e}}^{ - } + 4{\mathrm{H}}^{ + } + {\mathrm{O}}_{2} \to 2{\mathrm{H}}_{2} {\mathrm{O}}$$

2 $${\mathrm{The}}\;{\mathrm{total}}\;{\mathrm{stack}}\;{\mathrm{output}}\;{\mathrm{voltage}}\;{\mathrm{is}}:{\mathrm{V}}_{{{\mathrm{Stack}}}} = {\text{ N}}_{{{\mathrm{cell}}}} {\mathrm{V}}_{{{\mathrm{cell}}}} { }$$

The individual cell voltage is determined by four electrochemical components:

3 $${\mathrm{V}}_{{{\mathrm{cell}}}} = {\mathrm{E}}_{{{\mathrm{Nernst}}}} - {\mathrm{V}}_{{{\mathrm{act}}}} - {\text{ V}}_{{{\mathrm{ohm}}}} { } - {\text{ V}}_{{{\mathrm{con}}}} { }$$

The thermodynamic open-circuit voltage, corrected for temperature and reactant partial pressures, is given by the Nernst equation^9,48^:

4 $${\mathrm{E}}_{{{\mathrm{Nernst}}}} = 1.229 - 8.5 \times 10^{ - 4} \cdot \left( {{\mathrm{T}}_{{{\mathrm{FC}}}} - 298.15} \right) + 4.3085 \times 10^{ - 5} {\mathrm{T}}_{{{\mathrm{FC}}}} \left[ {{\mathrm{ln}}\left( {{\mathrm{P}}_{{{\mathrm{H}}_{2} }} } \right) + \frac{1}{2}{\mathrm{ln}}\left( {{\mathrm{P}}_{{{\mathrm{O}}_{2} }} } \right)} \right]$$

In Eq. (4) ${\mathrm{T}}_{{{\mathrm{FC}}}} { }$ is the operating temperature (K), and ${\mathrm{P}}_{{{\mathrm{H}}_{2} }}$ and ${\mathrm{P}}_{{{\mathrm{O}}_{2} }}$ are the effective partial pressures (atm) of hydrogen at the anode and oxygen at the cathode. The anode hydrogen partial pressure is^9^:

5 $${\mathrm{P}}_{{{\mathrm{H}}_{2} }} = 0.5 \cdot {\mathrm{RH}}_{{\mathrm{a}}} \cdot {\text{ P}}_{{{\mathrm{sat}}}} \cdot \left[ {\left( {\frac{{{\mathrm{e}}^{{\left( {1.635 - \frac{{{\mathrm{I}}_{{{\mathrm{FC}}}} }}{{\mathrm{A}}}} \right) \cdot {\mathrm{T}}_{{{\mathrm{FC}}}}^{ - 1.334} \cdot {\mathrm{RH}}_{{\mathrm{a}}} \cdot {\mathrm{P}}_{{{\mathrm{sat}}_{{{\mathrm{H}}_{2} {\mathrm{O}}}} }} { }}} }}{{{\mathrm{P}}_{{\mathrm{a}}} }}} \right)^{ - 1} - 1} \right]^{ - 1}$$

6 $${\mathrm{The}}\;{\mathrm{cathode}}\;{\mathrm{oxygen}}\;{\mathrm{partial}}\;{\mathrm{pressure}}\;{\mathrm{is}}:{\mathrm{P}}_{{{\mathrm{O}}2}} = \frac{{{\mathrm{RH}}_{{\mathrm{c}}} \cdot {\mathrm{P}}_{{{\mathrm{H}}_{2} {\mathrm{O}}}} }}{{\left( {\frac{{{\mathrm{P}}_{{\mathrm{c}}} }}{{{\mathrm{e}}^{{\left( {\frac{{4.192 \cdot {\mathrm{I}}_{FC} }}{{T_{FC}^{1.334} \cdot {\mathrm{A}}}}} \right) \cdot {\mathrm{RH}}_{{\mathrm{c}}} \cdot {\mathrm{P}}_{{{\mathrm{H}}_{2} {\mathrm{O}}}} }} }} - 1} \right)}}$$

The activation overpotential arises from the electrochemical reaction energy barrier at both electrodes. Following the Amphlett semi-empirical formulation^9^:

7 $${\mathrm{V}}_{{{\mathrm{act}}}} = - \left[ {{\upxi}_{1} + {\upxi}_{2} {\mathrm{T}}_{{{\mathrm{FC}}}} + {\upxi}_{3} {\mathrm{T}}_{{{\mathrm{FC}}}} {\mathrm{ln}}\left( {{\mathrm{C}}_{{{\mathrm{O}}_{2} }} } \right) + {\upxi}_{4} {\mathrm{T}}_{{{\mathrm{FC}}}} {\mathrm{ln}}\left( {{\mathrm{I}}_{{{\mathrm{FC}}}} } \right)} \right]$$

In Eq. (7), ${\mathrm{C}}_{{{\mathrm{O}}_{2} }}$ is the dissolved oxygen concentration at the cathode interface (mol/cm^3^), ${\mathrm{I}}_{{{\mathrm{FC}}}}$ is the fuel cell current (A), and ${\upxi}_{1}$, ${\upxi}_{2}$, ${\upxi}_{3}$, ${\upxi}_{4} { }$ are empirical coefficients that collectively govern the Tafel slope and exchange current density. The oxygen concentration is related to partial pressure by:

8 $${\mathrm{C}}_{{{\mathrm{O}}2}} = \frac{{{\mathrm{P}}_{{{\mathrm{O}}2}} }}{{5.08{ } \times { }10^{6} \cdot\exp \left( {{ }\frac{ - 1498}{{{\mathrm{T}}_{{{\mathrm{FC}}}} }}} \right)}}$$

The ohmic overpotential results from ionic resistance across the electrolyte membrane and electronic resistance in the external circuit^9,49^:

9 $${\mathrm{V}}_{{{\mathrm{ohm}}}} = {\mathrm{I}}_{{{\mathrm{FC}}}} \cdot \left( {{\mathrm{R}}_{{\mathrm{M}}} + {\mathrm{R}}_{{\mathrm{C}}} } \right)$$

The membrane ionic resistivity ${\uprho}_{{\mathrm{M}}}$ (kΩ·cm) is computed as:

10 $${\uprho}_{{\mathrm{M}}} = 181.6 \cdot \left( {\frac{{1 + 0.03 \cdot \frac{{{\mathrm{I}}_{{{\mathrm{FC}}}} }}{{\mathrm{A}}} + 0.062 \cdot \left( {\frac{{{\mathrm{T}}_{{{\mathrm{FC}}}} }}{303}} \right)^{2} \cdot \left( {\frac{{{\mathrm{I}}_{{{\mathrm{FC}}}} }}{{\mathrm{A}}}} \right)^{2.5} }}{{{\uplambda } - 0.634 - 3 \cdot \frac{{{\mathrm{I}}_{{{\mathrm{FC}}}} }}{{\mathrm{A}}}}}} \right) \cdot {\mathrm{e}}^{{\left( { - \frac{{4.18\left( {303 - {\mathrm{T}}_{{{\mathrm{FC}}}} } \right)}}{{{\mathrm{T}}_{{{\mathrm{FC}}}} }}} \right)}}$$

The membrane area-specific resistance is ${\text{ R}}_{{\mathrm{M}}} = {\uprho}_{{\mathrm{M}}} \frac{l}{A}{ }$, where ${\mathrm{l}}$ is the membrane thickness (cm) and A is the active area (cm^2^). The parameter λ represents the membrane water content and ${\mathrm{R}}_{{\mathrm{C}}} { }$ is the contact resistance (Ω).

Mass transport limitations at high current densities produce the concentration overpotential^9,50^:

11 $${\mathrm{V}}_{{{\mathrm{Con}}}} = - {\upbeta } \cdot {\mathrm{ln}}\left( {1 - \frac{{\mathrm{I}}}{{{\mathrm{A}} \cdot {\mathrm{J}}_{{{\mathrm{max}}}} }}} \right)$$

In Eq. (11) ${\upbeta }$ (V) is an empirical concentration coefficient,${\text{ J}}_{{{\mathrm{max}}}}$ (A/cm^2^) is the limiting current density, and A is the active membrane area (cm^2^). The stack output power is:

12 $${\mathrm{P}}_{{{\mathrm{Stack}}}} = {\mathrm{V}}_{{{\mathrm{Stack}}}} {\mathrm{I}}_{{{\mathrm{exp}}}}$$

### Parameter estimation problem formulation

The seven unknown parameters $X = \left[ { \xi_{1} , \xi_{2} , \xi_{3} , \xi_{4} , \lambda , \beta , R_{c} } \right]^{T}$ must be identified by minimising the sum of squared errors (SSE) between N experimentally measured and model-predicted terminal voltages^16,20,26^:

13 $${\mathrm{SSE}}\left( {\mathrm{X}} \right) = {\mathrm{min}}\left\{ {\mathop \sum \limits_{{{\mathrm{m}} = 1}}^{{\mathrm{n}}} \left( {{\mathrm{V}}_{{{\mathrm{exp}}}} \left( {\mathrm{m}} \right) - {\mathrm{V}}_{{{\mathrm{sim}}}} \left( {{\mathrm{m}},{\mathrm{X}}} \right)} \right)^{2} } \right\}$$

In Eq. (13) ${\mathrm{V}}_{{{\mathrm{exp}}}} \left( {\mathrm{m}} \right)$ is the experimental terminal voltage at the m^th^ current point and ${\mathrm{V}}_{{{\mathrm{sim}}}} \left( {{\mathrm{m}},{\mathrm{X}}} \right)$ is the model-predicted value computed from Eqs. (2)–(11). The implicit nonlinear current equation arising from Eq. (11) is resolved at each function evaluation using Newton–Raphson iteration. The optimization is subject to physical parameter bounds:

14 $${\mathrm{b}}_{{\mathrm{j}}}^{{{\mathrm{low}}}} \le {\mathrm{X}}_{{\mathrm{j}}} \le {\mathrm{b}}_{{\mathrm{j}}}^{{{\mathrm{high}}}} ,{\text{ j}} = 1,2, \ldots ,7$$

The search bounds adopted from the literature^9,13,16,26^ are listed in Table 2.

**Table 2:** EFTO and competitor algorithm parameter settings used in all experiments.

Algorithm	Parameter	Symbol	Value
All algorithms	Population size	N	50
	Maximum iterations	T	1000
	Independent runs	–	30
	Dimensionality (CEC 2017)	D	50
EFTO	Adaptive memory size	H	5
	Initial scaling factor memory	${\mathrm{M}}_{{{\mathrm{F}}\left( {\mathrm{h}} \right)}}$	0.30
	Initial crossover rate memory	${\mathrm{M}}_{{{\mathrm{CR}}\left( {\mathrm{h}} \right)}}$	0.80
	Scaling factor range	F	[0.0, 1.0]
	Crossover rate range	CR	[0.0, 1.0]
	Elite fraction	${\uprho}_{{\mathrm{e}}}$	0.15
	Elite guide weight	η	0.15
	Stagnation threshold	${\uptau}_{{{\mathrm{stag}}}}$	20
	QOBL trigger probability	${\mathrm{p}}_{{{\mathrm{QOBL}}}}$	0.40
	CAPS initial branch ratio	${\uprho}_{{\mathrm{c}}}$	0.25
	CAPS initial step size	σ	${\upsigma }$ = 0.3 ${\mathrm{mean}}\left( {{\mathrm{b}}_{{\mathrm{u}}} - {\mathrm{b}}_{{\mathrm{l}}} } \right)$
	ABP memory size	${\mathrm{H}}_{{{\mathrm{cl}}}}$	5
	ABP ratio bounds	$[{\uprho}_{{{\mathrm{min}}}} ,{\uprho}_{{{\mathrm{max}}}} ]$	[0.2, 0.8]
	M7 stall threshold	${\uptau}_{{{\mathrm{stall}}}}$	25
	M7 probe dimensions	${\mathrm{K}}_{{\mathrm{p}}}$	min(10, D)
	M8 trigger progress	${\upvarphi}_{{{\mathrm{M}}8}}$	φ > 0.92
	M8 evaluation budget	κ	15
SCA^51^	Constant for ${\mathrm{r}}_{1} { }$ decay	a	2
	Range of ${\mathrm{r}}_{2}$	${\mathrm{r}}_{2}$	[0,2π]
WOA^44^	Spiral shape constant	b	1
	Control parameter range	a	2 → 0 (linear)
	Spiral/shrink switch probability	*p*	0.5
GWO^52^	Control parameter range	a	2 → 0 (linear)
AOA^53^	Math optimiser probability min	${\mathrm{MOA}}_{{{\mathrm{min}}}}$	0.2
	Math optimiser probability max	${\mathrm{MOA}}_{{{\mathrm{max}}}}$	1.0
	Sensitive parameter	α	5
	Small integer	ε	0.1
MVO^54^	Min wormhole existence probability	${\mathrm{WEP}}_{{{\mathrm{min}}}}$	0.2
	Max wormhole existence probability	${\mathrm{WEP}}_{{{\mathrm{max}}}}$	1.0
	Travelling distance rate exponent	p	6
EDO^55^	Scaling factor	F	0.5
	Crossover rate	CR	0.9
LSHADE^28^	Memory size	H	6
	Min population size	${\mathrm{N}}_{{{\mathrm{min}}}}$	
jSO^29^	Memory size	H	
	Initial F memory	${\mathrm{M}}_{{\mathrm{F}}}$	0.3
Adaptive covariance search^30^	Initial step size	${\upsigma}_{0}$	0.3 $\left( {{\mathrm{b}}_{{\mathrm{u}}} - {\mathrm{b}}_{{\mathrm{l}}} } \right)$
	Parent number	${\upmu }$	N/2

## Proposed enhanced Fourier transform optimizer (EFTO)

The Enhanced Fourier Transform Optimizer (EFTO) is derived from the Fourier Transform Optimizer (FTO)^27^, which exploits Fourier transform principles to decompose signals into frequency components at different scales. Each candidate solution is transformed into the frequency domain using the DFT, where low-frequency components represent broad global structure and high-frequency components capture fine-grained local details. Following structured spectral operations, the modified solution is reconstructed using the inverse DFT. The parameter K_c_ decreases monotonically from D to 1, producing a coarse-to-fine spectral shrinkage process reminiscent of multigrid methods while naturally promoting the transition from exploration to exploitation without requiring additional parameter schedules. Unlike coordinate wise spatial operators, DFT-based recombination acts on the entire solution vector across all dimensions simultaneously, providing a geometrically distinct search mechanism in the solution space.

Instead of independent append-only modules, the eight mechanisms are specifically designed for FTO’s frequency-domain search topology and conditioned on the state of previous ones. M1 and M2 guarantee a good-spread, parameter-tuned initialization condition. M3 provides elite-guided exploitative mutation in solution space while M4 supplies rotation-invariant sampling in the covariance-adapted subspace, providing complementary search directions. M5 and M6 address stagnation recovery and boundary correction, and M7 and M8 apply precision refinement exclusively in the terminal phase.

The eight mechanisms of EFTO are designed around a principled exploration–exploitation (E/E) balance. Exploration is promoted by M1 (OBL initialization), M5 (quasi-oppositional stagnation recovery), and M7 (boundary exploration probe), while exploitation is progressively reinforced by M2 (adaptive parameter memory), M3 (elite-guided mutation), M4 (covariance-adaptive sampling), and M8 (terminal coordinate local search). M6 (midpoint boundary handling) operates as a corrective mechanism throughout. The exploration–exploitation (E/E) balance is managed at three levels. At the mechanism level, the balance is regulated through phase-aware activation conditions. At the branch level, the adaptive branch probability (ABP) mechanism continuously adjusts the selection probability of the two search branches according to the online success rates of the two search branches. At the population level, linear population size reduction (LPSR) progressively concentrates computational resources on the most promising individuals.

### Base FTO framework

The base FTO framework initializes a population (P) of N individuals uniformly at random over the D-dimensional search space bounded by ${\mathrm{b}}^{{\mathrm{l}}}$ and ${\mathrm{b}}^{{\mathrm{u}}}$^27^:

15 $${\mathrm{x}}_{{\mathrm{j}}}^{{\mathrm{n}}} = {\mathrm{b}}_{{\mathrm{j}}}^{{\mathrm{l}}} + {\mathrm{rand}}\left( {{\mathrm{b}}_{{\mathrm{j}}}^{{\mathrm{u}}} - {\mathrm{b}}_{{\mathrm{j}}}^{{\mathrm{l}}} } \right),{\text{ j}} = 1, \ldots ,{\mathrm{D}},{\text{ n}} = 1, \ldots .,{\mathrm{N}}$$

Each candidate ${\mathrm{x}}^{{\mathrm{n}}} \in {\mathrm{R}}^{{\mathrm{D}}}$ is transformed to the frequency domain via the DFT:

16 $$X_{k} = \mathop \sum \limits_{j = 0}^{D - 1} x_{j}^{n} \cdot e^{{\left( { - i\frac{2\pi kj}{D}} \right)}} ,\quad k = 0, 1,...,D - 1$$

Note: DFT indices are 0-based per convention; spatial component indices in Eq. (15) follow 1-based MATLAB convention.

The exploration phase applies Differential Frequency Mixing (DFM) using two randomly selected individuals r₁ ≠ r₂ ≠ n:

17 $$\Delta X = X^{r1} - X^{r2}$$

18 $$X_{new} \left( {1:K_{c} } \right) = X\left( {1:K_{c} } \right) + F \cdot \Delta X\left( {1:K_{c} } \right)$$

19 $$K_{c} = \max \left( {1,\;D \cdot \left( {1 - \frac{t}{T}} \right)} \right)[EFTO \, \;correction: \, ensures\;K_{c} \ge 1$$

20 $$step\left( {1:K_{c} } \right) = \frac{{u\left( {1:K_{c} } \right)}}{{\left| {v\left( {1:K_{c} } \right)} \right|^{{1/\beta_{L} }} }},u \sim {\mathcal{N}}\left( {0,\,\sigma_{L}^{2} } \right),v \sim {\mathcal{N}}\left( {0,\,1} \right)$$

21 $$X_{new} \left( {1:K_{c} } \right) = X\left( {1:K_{c} } \right) \cdot \left( {1 + \alpha \cdot step\left( {1:K_{c} } \right)} \right),\alpha = \frac{{\alpha_{0} }}{1 + 0.05\,t},\alpha_{0} = 0.1$$

where the notation ${\mathrm{X}}\left( {1:{\mathrm{K}}_{{\mathrm{c}}} } \right)$ denotes the sub-vector $\left[ {{\mathrm{X}}_{1} {\mathrm{X}}_{2} , \ldots {\mathrm{X}}_{{{\mathrm{K}}_{{\mathrm{c}}} }} } \right]$ comprising the first ${\mathrm{K}}_{{\mathrm{c}}}$ components.

An adaptive linear low-pass filter suppresses high-frequency components progressively:

22 $${\mathrm{cutoff}}\left( {\mathrm{t}} \right) = 0.9 - 0.8 \cdot \frac{{\mathrm{t}}}{{\mathrm{T}}}$$

23 $${\mathrm{H}}_{{\mathrm{k}}} = \left\{ {\begin{array}{*{20}l} 1 \hfill & {{\text{if NFG}}_{{\mathrm{k}}} \le {\mathrm{cutoff}}\left( {\mathrm{t}} \right)} \hfill \\ 0 \hfill & {{\mathrm{otherwise}}} \hfill \\ \end{array} } \right.;{\mathrm{X}}_{{{\mathrm{new}}}} = {\mathrm{X}}_{{\mathrm{k}}} \cdot {\mathrm{H}}_{{\mathrm{k}}}$$

where $${\mathrm{NFG}}_{{\mathrm{k}}} = {\raise0.7ex\hbox{${\mathrm{k}}$} \!\mathord{\left/ {\vphantom {{\mathrm{k}} {{\mathrm{D}} - 1}}}\right.\kern-0pt} \!\lower0.7ex\hbox{${{\mathrm{D}} - 1}$}}$$ denotes the normalized frequency of the k^th^ DFT component, k = 0,1,…,D − 1.

The inverse DFT (IDFT) reconstructs candidate solutions and elite retention replaces the worst ${\mathrm{n}}_{{\mathrm{e}}}$ = ⌊0.1·N⌋ individuals with elite members:

24 $${\hat{\mathrm{x}}}_{{\mathrm{k}}}^{{\mathrm{n}}} { } = { }\frac{1}{{\mathrm{D}}}\mathop \sum \limits_{{{\mathrm{j}}_{ = 0} }}^{{{\mathrm{D}} - 1}} {\mathrm{X}}_{{\mathrm{j}}} { }\cdot{\text{ exp}}\left( {{\text{i }}\cdot{ }2{\uppi }\frac{{{\mathrm{kj}}}}{{\mathrm{D}}}} \right){ }\left[ {{\mathrm{IDFT}}} \right]$$

25 $$\hat{x}^{i} \leftarrow \hat{x}^{elite} \left( i \right), i \in worst_{idx} , \left| {worst_{idx} } \right| = 0.1 \cdot N$$

**Figure:**
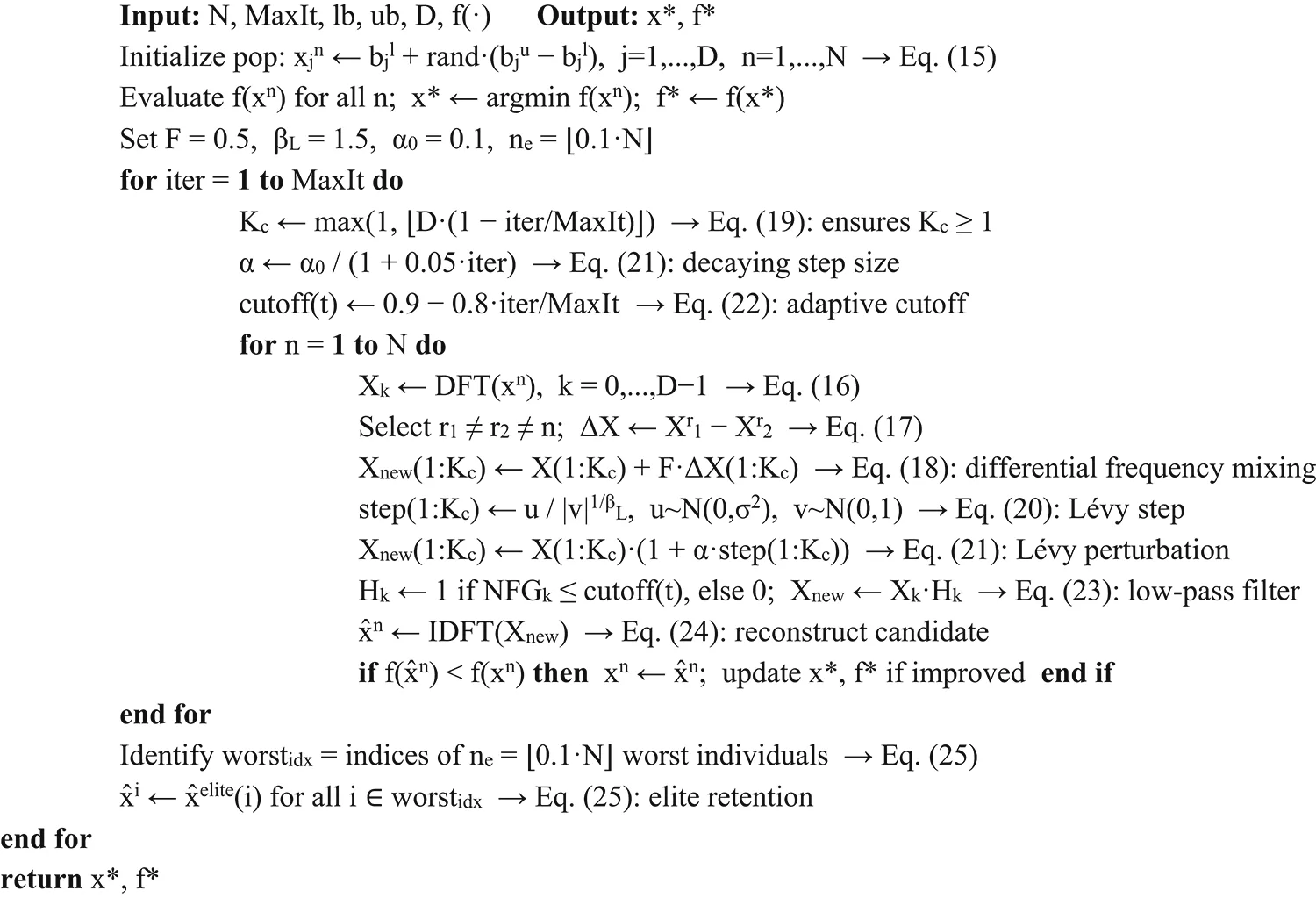
Algorithm-1 Base Fourier Transform Optimizer (FTO).

The base FTO framework exhibits three critical limitations. First, the hard binary spectral mask (Eq. 23) introduces discontinuous spectral transitions at the cutoff frequency, causing numerical instability on rotated high-dimensional functions. Second, the fixed scaling factor F = 0.5 (Eq. 18) is suboptimal across heterogeneous CEC 2017 landscapes spanning both unimodal and multimodal functions. Third, the absence of elite guidance in the DFM operator (Eqs. 17–18) causes slow convergence on multimodal functions with deceptive basins.

### Mechanism 1—opposition-based learning initialization

Standard random initialization (Eq. 15) provides no structural coverage guarantee across asymmetric, multi-scale parameter spaces. Let ${\mathrm{N}}_{{\mathrm{h}}} = \frac{{\mathrm{N}}}{2}$. The first half of the population is drawn uniformly at random and each candidate generates an opposing counterpart:

26 $${\tilde{\mathrm{x}}}_{{\mathrm{j}}}^{{\mathrm{n}}} { } = {\text{ max}}\left( {{\mathrm{b}}_{{\mathrm{j}}}^{{\mathrm{l}}} ,{\text{ min}}\left( {{\mathrm{b}}_{{\mathrm{j}}}^{{\mathrm{u}}} ,{\text{ b}}_{{\mathrm{j}}}^{{\mathrm{l}}} { } + {\text{ b}}_{{\mathrm{j}}}^{{\mathrm{u}}} { } - {\text{ x}}_{{\mathrm{j}}}^{{\mathrm{n}}} } \right)} \right),{\text{ j }} = { }1,{ } \ldots ,{\text{ D}}$$

The combined candidate pool ${\Xi } = \left\{ {{\mathrm{x}}^{1} ,{\mathrm{x}}^{2} ,{ } \ldots {\mathrm{x}}^{{{\mathrm{N}}_{{\mathrm{h}}} }} ,{\text{ x}}^{\sim 1} ,{\text{ x}}^{\sim 2} , \ldots .{\mathrm{x}}^{{\sim {\mathrm{N}}_{{\mathrm{h}}} }} } \right\}$ of size ${\mathrm{N}}_{{\mathrm{h}}}$ is formed and the ${\mathrm{N}}_{{\mathrm{h}}} { }$ best-fitness individuals are retained:

27 $$P_{EFTO} = \arg \min_{{\left| S \right| = N_{h} }} \left\{ { f\left( x \right) : x \in \Xi } \right\} \left[ {{\mathrm{retain}} \;{\mathrm{best}}\; N_{h} \;{\mathrm{from}}\; N \;{\mathrm{candidates}}} \right]$$

M1 directly addresses the first FTO deficiency: purely random initialization provides no systematic coverage of the asymmetric, constraint-bounded PEMFC parameter space. By evaluating both a random solution and the opposition of the random solution simultaneously, M1 doubles effective initial coverage without additional algorithmic complexity, statistically improving the probability of beginning the search in a productive feasible region.

### Mechanism 2—SHADE adaptive parameter control

EFTO maintains a circular memory buffer of size H = 5 (${\mathrm{M}}_{{\mathrm{F}}}$ and ${\mathrm{M}}_{{{\mathrm{CR}}}}$) initialised to ${\mathrm{M}}_{{{\mathrm{F}}\left( {\mathrm{h}} \right)}}$ = 0.30 and ${\mathrm{M}}_{{{\mathrm{CR}}\left( {\mathrm{h}} \right)}}$ = 0.80 for h = 1,…,5. At each iteration, F and CR are sampled from Cauchy and Normal distributions respectively:

28 $${\text{F }} = {\text{ clip}}\left( {{\mathrm{M}}_{{\mathrm{F}}} \left( {\mathrm{r}} \right){ } + { }0.1{ }\cdot{\text{ tan}}\left( {{\pi }\cdot{ }\left( {{\text{rand }} - { }0.5} \right)} \right),{ }0,{ }1.0} \right),{\text{ r }}\sim {\text{ U}}\left\{ {1,{ } \ldots ,{\text{ H}}} \right\}$$

where clip(x, a, b) = max(a, min(b, x)) denotes element-wise clamping to [a, b].

29 $${\text{CR }} = {\text{ clip}}\left( {{\mathrm{M}}_{{{\mathrm{CR}}}} \left( {\mathrm{r}} \right){ } + { }0.1{ }\cdot{\text{ N}}\left( {0,{ }1} \right),{ }0,{ }1} \right)$$

A progress-aware factor F̃ adapts the p-best attraction strength based on $${\upvarphi }\left( {\mathrm{t}} \right) = {\raise0.7ex\hbox{${{\mathrm{NFE}}}$} \!\mathord{\left/ {\vphantom {{{\mathrm{NFE}}} {{\mathrm{NFE}}_{{{\mathrm{max}}}} }}}\right.\kern-0pt} \!\lower0.7ex\hbox{${{\mathrm{NFE}}_{{{\mathrm{max}}}} }$}}:$$

30 $$\tilde{F} = \min \left( {1.2, F \times \gamma \left( \varphi \right)} \right), \gamma \left( \varphi \right) = \{ 0.7\;{\mathrm{if}}\;\varphi < 0.2; 0.8\;{\mathrm{if}}\;\varphi < 0.4; 1.2\;{\mathrm{otherwise}})$$

Memory updates use fitness-improvement-weighted Lehmer means at each iteration end:

31 $${\mathrm{w}}_{{\mathrm{s}}} { } = { }\frac{{{\Delta f}_{{\mathrm{s}}} { }}}{{\mathop \sum \nolimits_{{\mathrm{i}}} {\Delta f}_{{\mathrm{i}}} }};{\text{ M}}_{{\mathrm{F}}} \left( {{\mathrm{ptr}}} \right){ } \leftarrow { }\frac{{\mathop \sum \nolimits_{{\mathrm{s}}} {\mathrm{w}}_{{\mathrm{s}}} { }\cdot{\text{ F}}_{{\mathrm{s}}}^{2} }}{{\mathop \sum \nolimits_{{\mathrm{s}}} {\mathrm{w}}_{{\mathrm{s}}} { }\cdot{\text{ F}}_{{\mathrm{s}}} }};{\text{ M}}_{{{\mathrm{CR}}}} \left( {{\mathrm{ptr}}} \right){ } \leftarrow { }\mathop \sum \limits_{{\mathrm{s}}} {\mathrm{w}}_{{\mathrm{s}}} { }\cdot{\text{ CR }}_{{\mathrm{s}}}$$

M2 directly addresses the second FTO deficiency: the fixed scaling factor F = 0.5 cannot adapt to the evolving search landscape. By updating the F and CR parameters using successful trial solutions through a weighted Lehmer mean, M2 gradually adjusts the exploration–exploitation balance during the search. Larger exploratory moves are favored in the early stages, whereas more exploitative behavior emerges later based on the success of previously accepted solutions.

### Mechanism 3—elite-guided mutation with diversity archive

EFTO maintains an elite set ${\mathcal{E}}$, consisting of the top ${\uprho}_{{\mathrm{e}}} = 15{{\% }}$ individuals, and a diversity archive containing replaced solutions with a maximum capacity of ∣∣ = N. The population centroid is computed as:

32 $${\tilde{\mathrm{x}}} = \frac{1}{{\mathrm{N}}}\mathop \sum \limits_{{{\mathrm{n}} = 1}}^{{\mathrm{N}}} {\mathrm{x}}^{{\mathrm{n}}}$$

The size of the p-best pool is adaptively adjusted according to the search progress ϕ(t):

32a $${\mathrm{N}}_{{\mathrm{p}}} = {\mathrm{max}}\left( {2,{\text{ round}}\left[ {{\mathrm{N}}.{\text{ max}}\left( {0.05,{ }0.25\left( {1 - 0.5\phi \left( {\mathrm{t}} \right)} \right)} \right)} \right]} \right)$$

The mutation vector combines four directional components:

33 $$v^{n} = x^{n} + \tilde{F} \cdot \left( {x^{{\left( {pb} \right)}} - x^{n} } \right) + F \cdot \left( {x^{r1} - x^{r2} } \right) + 0.15 \cdot \left( {x^{\left( g \right)} - \overline{x}} \right)$$

where ${\mathrm{p}}_{{\mathrm{b}}} \sim {\mathrm{U}}\left\{ {1, \ldots .,{\mathrm{N}}_{{\mathrm{p}}} } \right\}$ denotes the selected p-best index, ${\mathrm{r}}_{1} \sim {\mathrm{U}}\left\{ {1, \ldots .,{\mathrm{N}}_{{\mathrm{p}}} } \right\}\backslash \left\{ {\mathrm{n}} \right\}$ is a randomly selected population index excluding the current individual, ${\mathrm{r}}_{2}$. is selected from the augmented pool ${\mathrm{P}} \cup { }{\mathcal{A}}$ and ${\mathrm{g}}\sim \cup \left\{ {1, \ldots .,{\mathrm{n}}_{{\mathrm{c}}} } \right\}$ represents the elite guide index. Binomial crossover is then applied to generate the trial vector:

34 $$u_{j}^{n} = \{ v_{j}^{n} \;{\mathrm{if}}\;{\text{rand }}_{j} \le CR \;{\mathrm{or}}\; j = j_{rand} , {\mathrm{else}}\; x_{j}^{n} ), j = 1, \ldots , D$$

where ${\mathrm{j}}_{{{\mathrm{rand}}}} \sim {\mathrm{U}}\left\{ {1, \ldots ..,{\text{ D}}} \right\}$ is a uniformly sampled mandatory crossover index ensuring ${\mathrm{u}}^{{\mathrm{n}}} \ne {\mathrm{x}}^{{\mathrm{n}}}$.

Upon successful improvement, the parent solution ${\mathrm{x}}^{{\mathrm{n}}}$ is stored in the diversity archive . If the archive size exceeds its maximum capacity (∣ ∣ > N), a randomly selected archive member is replaced.

The elite-guided component 0.15·(xᵍ − x̅) augments the standard current-to-best perturbation with a directional pull toward the population consensus region, increasing exploitation intensity in a manner that scales naturally with population quality as the elite centroid becomes increasingly accurate across iterations.

### Mechanism 4—covariance-adaptive population sampling (CAPS)

The hard binary spectral mask of the base FTO (Eq. 23) is unable to adapt to rotated or ill-conditioned search landscapes. Mechanism 4 introduces Covariance-Adaptive Population Sampling (CAPS), which creates candidates along the local fitness geometry by estimating the covariance of a subset of top performers. The original FFT recombination was deleted after CEC2017 benchmarking, since the original FFT recombination became unstable numerically on high-dimensional rotated functions (F1 = 7.20 × 10^3^ with FFT; reduced to F1 = 100 when replaced). CAPS runs on a fraction of the population (initially set to 0.25). Let be a covariance matrix, B the eigenvector matrix of the covariance matrix, and the vector of square-root eigenvalues. Each CAPS candidate is sampled as:

35 $$z \sim N\left( {0, I_{D} } \right); u^{n} = \mu + \sigma \cdot B \cdot \left( {\Lambda \odot z} \right)$$

where μ is the weighted mean of the top ${\upmu}_{{\mathrm{w}}}$ = ⌊N/2⌋ elite individuals, σ > 0 is the global step size, and ⊙ denotes element-wise multiplication. The recombination weights are computed as

$$w_{i} = \frac{{\ln \left( {\mu_{w} + 0.5} \right) - \ln \left( i \right)}}{{\mathop \sum \nolimits_{j} \ln \left( {\mu_{w} + 0.5} \right) - \ln \left( j \right)}}$$

Such that the normalized weights satisfy $\mathop \sum \limits_{{\mathrm{i}}} {\mathrm{w}}_{{\mathrm{i}}} = 1$.

Where ${\upmu}_{{{\mathrm{eff}}}} = \mathop \sum \limits_{{\mathrm{i}}} {\mathrm{w}}_{{\mathrm{i}}}^{2}$ denotes the effective selection mass, quantifying the diversity of the recombination weight distribution.

Two cumulative evolution paths ${\mathrm{p}}_{{\upsigma }} {\text{ and p}}_{{\mathrm{c}}}$ track the search trajectory:

36 $$p_{\sigma } \leftarrow \left( {1 - c_{\sigma } } \right) \cdot p_{\sigma } + \sqrt {c_{\sigma } \cdot \left( {2 - c_{\sigma } } \right) \cdot \mu_{eff} } \cdot C^{ - 1/2} \cdot \frac{{\mu - \mu_{old} }}{\sigma }$$

37 $$p_{c} \leftarrow \left( {1 - c_{c} } \right) \cdot p_{c} + h_{sig} \cdot \sqrt {c_{c} \cdot \left( {2 - c_{c} } \right) \cdot \mu_{eff} } \cdot \frac{{\mu - \mu_{old} }}{\sigma }$$

$$h_{sig} = \left\{ {\begin{array}{*{20}l} { - 1,} \hfill & {if\frac{{\left\| {p_{\sigma } } \right\|}}{{\chi N \sqrt {1 - \left( {1 - c_{\sigma } } \right)^{\frac{2NFE}{N}} } }} < 1.4 + \frac{2}{D + 1},} \hfill \\ {0,} \hfill & {otherwise.} \hfill \\ \end{array} } \right.$$

The covariance matrix and step size is updated as:

38 $${\text{C }} \leftarrow { }\left( {1{ } - {\text{ c}}_{1} { } - {\text{ c}}_{{\upmu }} } \right){ }\cdot{\text{ C }} + {\text{ c}}_{1} { }\cdot{\text{ p}}_{{\mathrm{c}}} { }\cdot{\text{ p}}_{{\mathrm{c}}}^{{\mathrm{T}}} { } + {\text{ c}}_{{\upmu }} { }\cdot{ }\mathop \sum \limits_{{\mathrm{i}}} {\mathrm{w}}_{{\mathrm{i}}} { }\cdot{\text{ a}}_{{\mathrm{i}}} { }\cdot{\text{ a}}_{{\mathrm{i}}}^{{\mathrm{T}}} { }$$

39 $$\sigma \leftarrow \sigma \cdot \exp \left( {\frac{{c_{\sigma } }}{{d_{\sigma } }} \cdot \left( {\frac{{p_{\sigma } }}{{\chi^{N} }} - 1} \right)} \right)$$

$${\mathrm{c}}_{{\mathrm{c}}} = \frac{{\left( {4 + {\raise0.7ex\hbox{${{\upmu}_{{{\mathrm{eff}}}} }$} \!\mathord{\left/ {\vphantom {{{\upmu}_{{{\mathrm{eff}}}} } {\mathrm{D}}}}\right.\kern-0pt} \!\lower0.7ex\hbox{${\mathrm{D}}$}}} \right)}}{{\left( {{\mathrm{D}} + 4 + {\raise0.7ex\hbox{${2{\upmu}_{{{\mathrm{eff}}}} }$} \!\mathord{\left/ {\vphantom {{2{\upmu}_{{{\mathrm{eff}}}} } {\mathrm{D}}}}\right.\kern-0pt} \!\lower0.7ex\hbox{${\mathrm{D}}$}}} \right)}},{\text{ c}}_{{\upsigma }} = \frac{{\left( {{\upmu}_{{{\mathrm{eff}}}} + 2} \right)}}{{\left( {{\mathrm{D}} + {\upmu}_{{{\mathrm{eff}}}} + 5} \right)}}$$

$${\mathrm{a}}_{{\mathrm{i}}} = \frac{{{\mathrm{x}}^{{\left( {\mathrm{i}} \right)}} - {\upmu}_{{{\mathrm{old}}}} }}{{\upsigma }},{\text{ c}}_{1} = \frac{2}{{\left( {{\mathrm{D}} + 1.3} \right)^{2} + {\upmu}_{{{\mathrm{eff}}}} }},{\text{ c}}_{{\upmu }} = {\mathrm{min}}\left( {1 - {\mathrm{c}}_{1} ,{ }\frac{{2\left( {{\upmu}_{{{\mathrm{eff}}}} - 2 + \frac{1}{{{\upmu}_{{{\mathrm{eff}}}} }}} \right)}}{{\left( {{\mathrm{D}} + 2} \right)^{2} + {\upmu}_{{{\mathrm{eff}}}} }}} \right)$$

$$d_{\sigma } = 1 + 2 \max \left( {0, \sqrt {\frac{{\mu_{eff} - 1}}{D + 1}} - 1} \right)c_{\sigma } , \chi_{N} = \sqrt D \left( {1 - \frac{1}{4D} + \frac{1}{{21D^{2} }}} \right)$$

The fraction ${\uprho}_{{\mathrm{c}}} { }$ adapts via Adaptive Branch Probability (ABP) based on per-iteration success rates of the CAPS and DE branches:

40 $$\rho_{c} \leftarrow 1 - mean\left( {M_{cl} } \right), M_{cl} \;updated\; by: p_{new} = clip\left( {\frac{{r_{DE} }}{{r_{DE} + r_{CAP} + \varepsilon }}, 0.2, 0.8} \right)$$

M4 addresses the third FTO deficiency: the original frequency-domain operator becomes inactive in the final 14.2% of the run and cannot model variable correlations. CAPS replaces the original frequency-domain operator with a covariance-matrix-based sampling distribution that learns the landscape correlation structure through rank-μ updates, enabling rotation-invariant exploitation particularly effective for the non-separable PEMFC SSE objective.

### Mechanism 5—stagnation-triggered quasi-opposition recovery

An individual n is considered stagnated (counter ${\upvarepsilon}_{{\mathrm{n}}}$) when it fails to improve for ${\uptau}_{{{\mathrm{stag}}}} = 20{ }$ consecutive iterations. When ${\upvarepsilon}_{{\mathrm{n}}}$ > 20, rand < 0.40, and φ(t) < 0.60, a quasi-opposite jump is applied:

41 $$\tilde{x}_{j} = b_{j}^{l} + b_{j}^{u} - u_{j}^{n} ; lo_{j} = \min \left( {u_{j}^{n} , \tilde{x}_{j} } \right); hi_{j} = \max \left( {u_{j}^{n} , \tilde{x}_{j} } \right)$$

42 $$u_{j}^{n} \leftarrow lo_{j} + rand _{j} \cdot \left( {hi_{j} - lo_{j} } \right), j = 1, \ldots , D$$

Quasi-opposition recovery (Eqs. 41–42) is activated exclusively when D ≤ 30, as at higher dimensions such as D = 50, the probability that a quasi-opposite point lies closer to the optimum decreases exponentially with dimension, rendering the operator ineffective. Deactivating M5 at D > 30 thus preserves the evaluation budget for the more effective high-dimensional mechanisms M3 and M4, which remain active at all dimensionalities.

M5 addresses the fourth FTO deficiency: the complete absence of any stagnation recovery strategy. Instead of reinitializing randomly, QOBL produces a quasi-opposite point in a statistically complementary region, presenting an escape with directionality of higher probability of finding progress than a random jump. D ≤ 30 and progress < 0.60 restrict M5 activation to the exploration-dominant phase.

### Mechanism 6—midpoint boundary handling

Infeasible trial vectors are mapped back using midpoint reflection:

43 $$u_{j}^{n} \leftarrow \frac{{b_{j}^{l} + x_{j}^{n} }}{2} \; if\; u_{j}^{n} \left\langle { b_{j}^{l} ; u_{j}^{n} \leftarrow \frac{{b_{j}^{u} + x_{j}^{n} }}{2}\; if\; u_{j}^{n} } \right\rangle b_{j}^{u}$$

44 $${\mathrm{u}}_{{\mathrm{j}}}^{{\mathrm{n}}} { } \leftarrow {\text{ max}}\left( {{\mathrm{b}}_{{\mathrm{j}}}^{{\mathrm{l}}} ,{\text{ min}}\left( {{\mathrm{b}}_{{\mathrm{j}}}^{{\mathrm{u}}} ,{\text{ u}}_{{\mathrm{j}}}^{{\mathrm{n}}} } \right)} \right),{\text{ j }} = { }1,{ } \ldots ,{\text{ D }}\left[ {\text{feasibility guarantee}} \right]$$

M6 tackles the fifth FTO deficiency: hard boundary clipping induces spectral discontinuities, which corrupt DFT-based perturbation quality. M6 preserves neighborhood information and smooth spectral properties essential for accurate frequency-domain search, by placing violated components at the midpoint between the parent value and the violated bound.

### Mechanism 7—budget-aware boundary probe

The boundary probe activates only when ${\mathrm{stall}}_{{{\mathrm{cnt}}}} > 25$ and NFE + 20 ≤ ${\mathrm{NFE}}_{{{\mathrm{max}}}}$, selecting ${\mathrm{K}}_{{\mathrm{p}}} = {\mathrm{min}}\left( {10,{\mathrm{D}}} \right)$ random dimensions:

45 $$x_{j}^{lo} = x* with x* _{j} = b_{j}^{l} ; x_{j}^{hi} = x* with x* _{j} = b_{j}^{u} , j \in {\mathcal{I}} _{p} , \left| {{\mathcal{I}} _{p} } \right| = K_{p}$$

46 $${\text{x* }} \leftarrow {\text{ argmin}}_{{{\text{x }} \in { }\left\{ {{\mathrm{x}}_{{\mathrm{j}}}^{{{\mathrm{lo}}}} ,{\text{ x}}_{{\mathrm{j}}}^{{{\mathrm{hi}}}} ,{\text{ x*}}} \right\}}} {\mathrm{f}}\left( {\mathrm{x}} \right),{ }\forall {\text{ j }} \in { }{\mathcal{I}}{ }_{{\mathrm{p}}} { }\left[ {\text{greedy acceptance}} \right]$$

Similarly, the boundary probe (Eqs. 45–46) is deactivated at D > 30 because at high dimensions the marginal contribution becomes too expensive since the boundary probe evaluates Kₚ whenever activated. However, note that the coordinate local search (Eqs. 47–48) is always active at any dimensionality since the adaptive step size δⱼ(t) of the coordinate local search scales naturally with the bounds of each search space dimension regardless of problem dimension.

To mitigate global population stagnation, M7 focuses on the constraint boundary, a vital area for PEMFC parameters estimation as optimal values of Rc and β commonly converge to the boundaries of Rc and β according to Table 7. M7 only activates after global stagnation has been confirmed (stall > 25 iterations), ensuring M7 does not consume NFE budget during productive search phases.

### Mechanism 8—terminal-phase coordinate local search

Coordinate local search applies exclusively in the terminal phase (φ(t) > 0.92, every 25 iterations, ≤ 15 evaluations per activation):

47 $$\delta_{j} \left( t \right) = \left( {0.25 \cdot \left( {1 - \varphi \left( t \right)} \right) + 0.003} \right) \cdot \left( {b_{j}^{u} - b_{j}^{l} } \right), j = 1, \ldots , D$$

48 $$x* \leftarrow \arg \min_{{x \in \left\{ {x* + \delta_{j} \cdot e_{j} , x* - \delta_{j} \cdot e_{j} , x*} \right\}}} f\left( x \right); \delta_{j} \leftarrow 0.5 \cdot \delta_{j} \; if \;no\; improvement$$

where ${\mathrm{j}} \in {\mathrm{R}}^{{\mathrm{D}}}$ is the j-th standard basis vector with 1 in position j and 0 elsewhere.

Terminal-phase activation (progress > 0.92) guarantees M8 refines an already high-quality solution instead of filtering the search too early. At κ = 15 evaluations per activation, M8 uses less than 0.03% of the total NFE budget and the computational cost of M8 is indeed asymptotically negligible in terms of the obtained precision value.

### Linear population size reduction (LPSR)

EFTO reduces N linearly with evaluation expenditure from ${\mathrm{N}}_{{{\mathrm{max}}}}$ to ${\mathrm{N}}_{{{\mathrm{min}}}}$ = 4:

49 $${\mathrm{N}}\left( {{\mathrm{NFE}}} \right){ } = {\text{ max}}\left( {{\mathrm{N}}_{{{\mathrm{min}}}} ,{\text{ round}}\left( {\frac{{\left( {{\mathrm{N}}_{{{\mathrm{min}}}} { } - {\text{ N}}_{{{\mathrm{max}}}} } \right)}}{{{\mathrm{NFE}}_{{{\mathrm{max}}}} }}{ }\cdot{\text{ NFE }} + {\text{ N}}_{{{\mathrm{max}}}} } \right)} \right)$$

where $${\text{ N}}_{{{\mathrm{max}}}} = {\mathrm{N}}\left( {\text{initial population size}} \right){\text{and N}}_{{{\mathrm{min}}}} = 4$$.

### Convergence properties

Although formal convergence proofs for stochastic population-based optimizers remain an open research problem, EFTO adheres to two practical convergence rules. (1) Monotone improvement. Both mechanisms M7 and M8 use greedy acceptance (Eqs. 46 and 48) that guarantees ${\mathrm{f}}\left( {{\mathrm{x}}^{{{*}\left( {{\mathrm{t}} + 1} \right)}} } \right) \le {\mathrm{f}}\left( {{\mathrm{x}}^{{\mathrm{*t}}} } \right)$ across all iterations t. (2) Asymptotic population compression. As ${\mathrm{NFE}} \to {\mathrm{NFE}}_{{{\mathrm{max}}}}$, LPSR reduces ${\text{N from N}}_{{{\mathrm{max}}}} {\text{ to N}}_{{{\mathrm{min}}}} = 4$, concentrating the final search in an ever-decreasing neighborhood of the found optimum simulating an annealing-like cooling schedule.

### Exploration–exploitation optimization

EFTO optimizes the exploration–exploitation balance through three self-adaptive mechanisms without requiring user-defined phase schedules. At the branch level, the ABP mechanism tracks per-iteration success rates of the jSO branch (r₁ = c₁,suc/c₁,tot) and CMA branch (r₂ = c₂,suc/c₂,tot), updating ρc ← 1 − mean(Mcl), where Mcl stores clip(r₁/(r₁ + r₂), 0.2, 0.8); bounds [0.2, 0.8] ensure neither branch is fully eliminated. At the parameter level, the SHADE memory continuously narrows the effective F and CR distributions toward values associated with improvement, automatically transitioning from exploration to exploitation as learning accumulates. At the population level, LPSR reduces N linearly from N_max_ = 50 to N_min_ = 4, progressively increasing selection pressure. Collectively, these three mechanisms adapt the exploration–exploitation balance according to observed search performance, eliminating the need for manually predefined scheduling strategies.

### Parameter selection justification and generalizability

The EFTO parameters settings in Table 2 are based on three design principles. First, self-adaptivity: F, CR and ρc are not fixed but adapted online by M2 (SHADE memory, Eqs. 28–31) as well as the ABP mechanism (Eq. 40), i.e. only the early stages of the search are affected by the initial memory values. Second, literature grounding: fixed-value parameters are set based on established conventions. Concretely, MF(h) = 0.30 and H = 5 adhere to the design of jSO^29^, meanwhile σ₀ = 0.3·mean(ub − lb) and μ = ⌊N/2⌋ implement the adaptive covariance search^30^. Third, dimension-aware deactivation: M5 and M7 get automatically deactivated when D > 30 without a need for problem-specific tuning. Notably, the same parameter settings were applied to both the CEC 2017 benchmark suite (D = 50, 28 functions) and PEMFC parameters estimation (D = 7) without any problem-specific fine-tuning.

**Figure:**
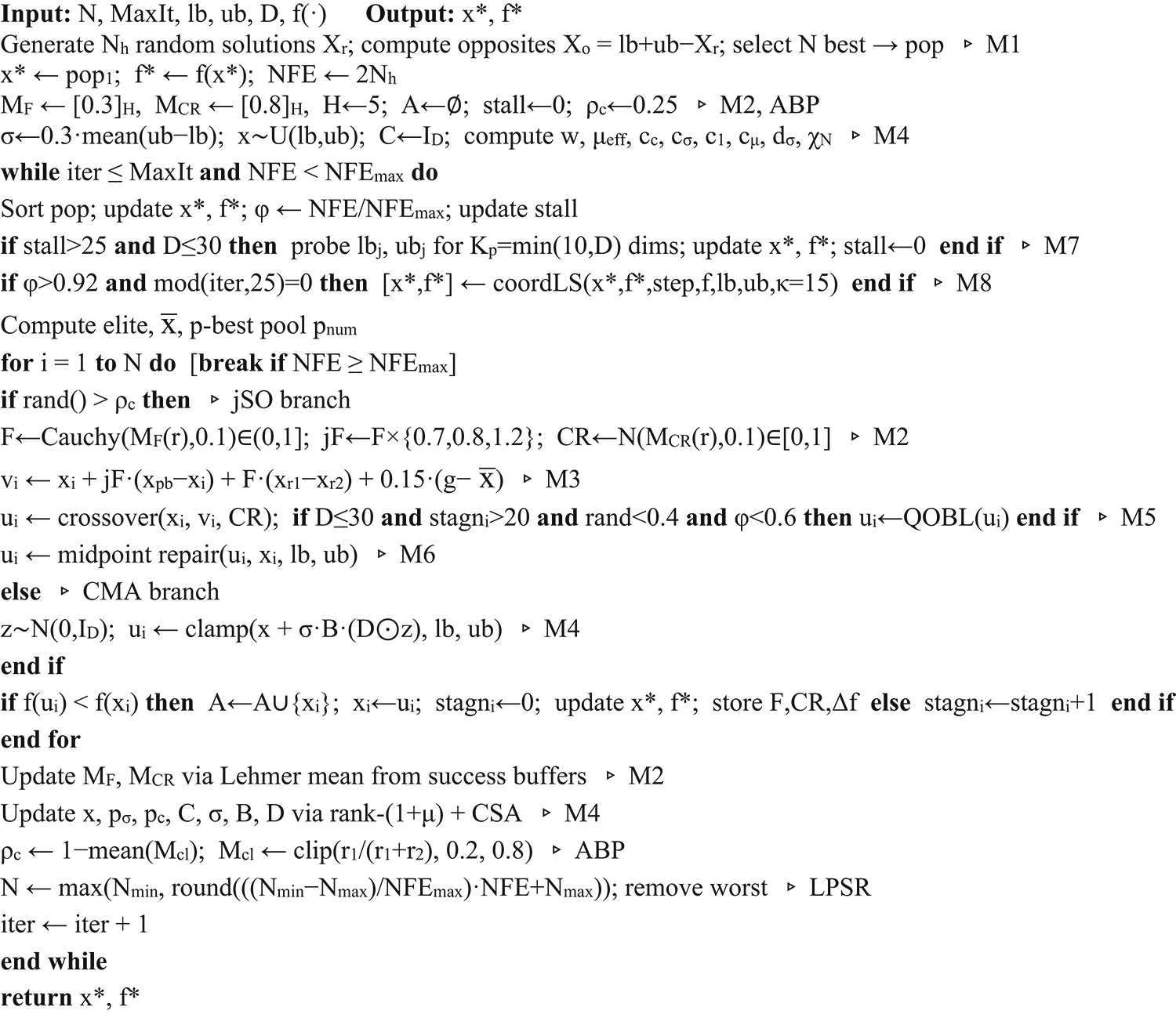
Algorithm-2 Enhanced Fourier Transform Optimizer (EFTO).

### Application to PEMFC parameter estimation

**Figure 1:**
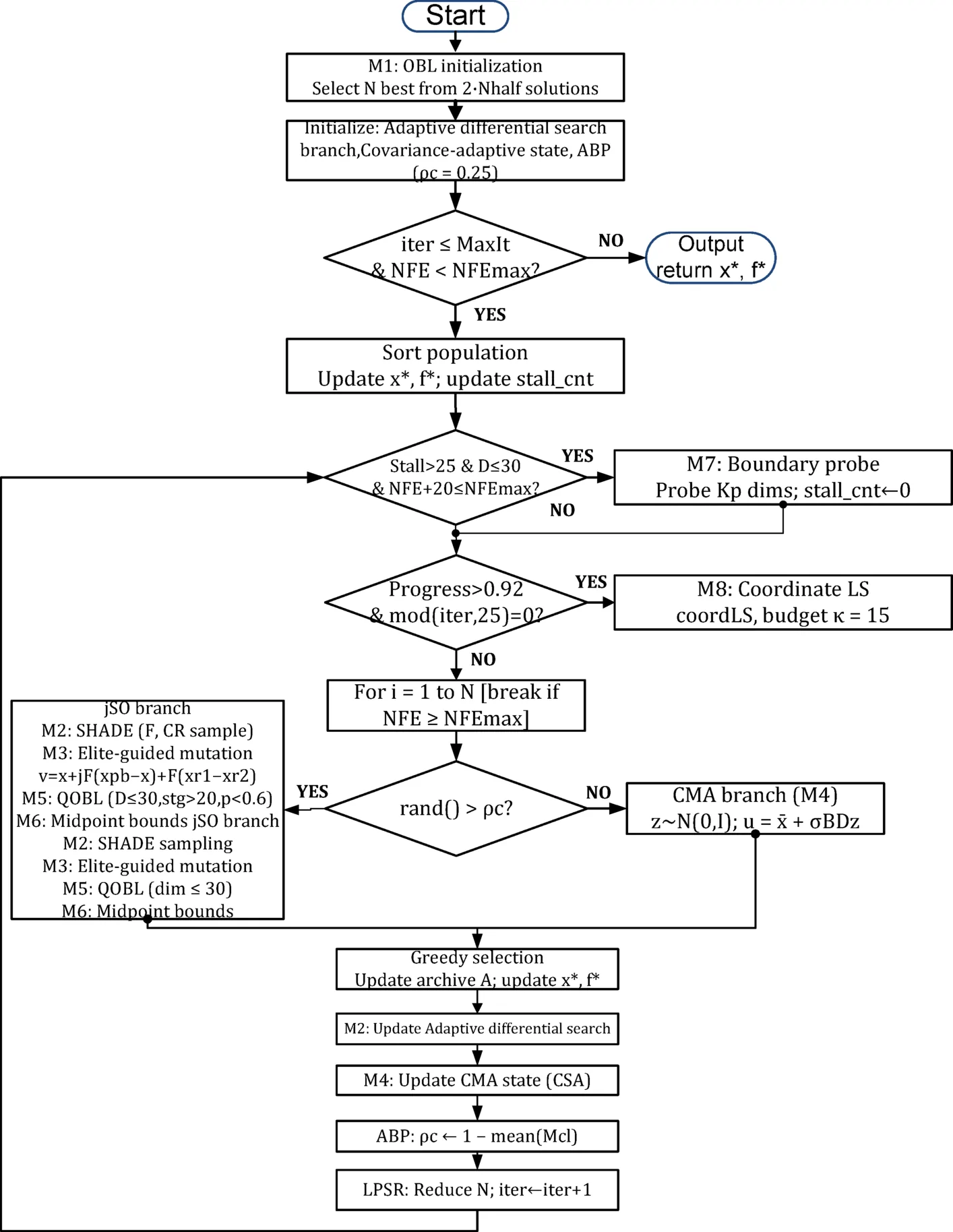
Flowchart of the proposed EFTO algorithm.

The EFTO-based PEMFC parameters extraction procedure is executed in five steps. Step 1: Set the decision variable dimension D = 7 (parameters[$\xi_{1} , \xi_{2} , \xi_{3} , \xi_{4} , \lambda , \beta , R_{c}$], population size ${\mathrm{N}}_{{{\mathrm{pop}}}}$ = 50, and maximum iterations MaxIt = 1000 per Table 4. Step 2: Evaluate the SSE objective function (Eq. 13) at each candidate solution by solving the implicit PEMFC current equation via Newton–Raphson iteration. Step 3: Execute EFTO (Algorithm 1, Fig. 1) over MaxIt iterations. Step 4: Record the optimal parameters vector ${\mathrm{X}}_{{{\mathrm{best}}}}$ and minimum SSE. Step 5: Reconstruct I-V and I-P curves from ${\mathrm{X}}_{{{\mathrm{best}}}} { }$ and compare against experimental data.

## Results and discussion

### Experimental setup and reproducibility

All experiments were conducted in MATLAB R2020b on a Windows 10 laptop equipped with an Intel Core i7 processor (2.6 GHz) and 16 GB RAM. Each algorithm was evaluated over 30 independent Monte Carlo runs using seeds 1–30 to ensure reproducibility. Population size and maximum iterations were fixed at N = 50 and MaxIt = 1,000 respectively for all algorithms and experiments, as listed in Table 4, yielding a total budget of NFE_max_ = 50,000 function evaluations per run. The CEC 2017 benchmark suite comprising 28 functions (F1 and F3–F29, excluding F2 as specified by the competition protocol) was employed for evaluation at D = 50 and D = 100. For PEMFC parameters estimation, the objective function is the sum of squared errors (SSE) between experimental and model-predicted terminal voltages, computed using the Amphlett semi-empirical model (Section "PEMFC mathematical model"). All comparison algorithm parameters were set as specified in the respective original publications of each algorithm.

### CEC 2017 benchmark validation

EFTO was evaluated on the IEEE CEC 2017 benchmark suite^56^ comprising 28 test functions across unimodal (F1, F3), multimodal (F4–F10), hybrid (F11–F20), and composition (F21–F29) categories at D = 50. EFTO was compared against SCA^51^, WOA^44^, AOA^53^, GWO^13^, MVO^54,57^, and EDO^55^ over 30 independent runs. Full statistical results (Mean ± Std) are shown in Table 3; Friedman rank and Wilcoxon signed-rank test results are summarized in Table 4.

**Table 3:** Statistical results (Mean ± Std) obtained on the CEC 2017 benchmark functions at D = 50 over 30 independent runs.

F	Type	EFTO	SCA	WOA	AOA	GWO	MVO	EDO
F1	Unimodal	**1.00e+02 ± 1.66e-04**	6.21e+10 ± 1.04e+10	4.02e+09 ± 8.17e+07	1.10e+11 ± 1.05e+10	8.80e+09 ± 2.12e+09	2.89e+06 ± 1.90e+05	3.83e+10 ± 4.78e+09
F3	Unimodal	**1.50e**+**03 ± 7.89e**+**02**	1.81e+05 ± 2.45e+03	2.06e+05 ± 9.12e+04	1.57e+05 ± 3.00e+04	1.11e+05 ± 6.86e+03	1.61e+04 ± 8.71e+03	1.05e+05 ± 1.34e+04
F4	Multimodal	**4.96e**+**02 ± 5.02e**+**01**	9.23e+03 ± 1.76e+03	1.92e+03 ± 8.39e+02	2.88e+04 ± 1.55e+04	1.77e+03 ± 1.14e+03	5.69e+02 ± 5.50e+01	8.50e+03 ± 9.81e+01
F5	Multimodal	**5.82e**+**02 ± 1.98e**+**01**	1.12e+03 ± 2.26e+01	1.04e+03 ± 1.12e+02	1.17e+03 ± 8.53e+00	7.14e+02 ± 3.69e+01	6.72e+02 ± 4.95e+01	1.07e+03 ± 4.38e+01
F6	Multimodal	**6.01e**+**02 ± 1.36e**+**00**	6.81e+02 ± 3.99e+00	7.04e+02 ± 5.81e+00	6.92e+02 ± 5.23e+00	6.17e+02 ± 1.16e+01	6.44e+02 ± 1.83e+00	6.76e+02 ± 3.57e+00
F7	Multimodal	**8.13e**+**02 ± 1.15e**+**01**	1.76e+03 ± 2.31e+01	1.83e+03 ± 1.88e+02	1.93e+03 ± 7.99e+01	1.12e+03 ± 8.28e+01	1.05e+03 ± 2.59e+01	1.60e+03 ± 6.03e+01
F8	Multimodal	**8.82e**+**02 ± 1.55e**+**01**	1.38e+03 ± 1.80e+01	1.32e+03 ± 4.18e+01	1.48e+03 ± 7.35e+01	1.03e+03 ± 1.61e+01	1.11e+03 ± 7.13e+01	1.37e+03 ± 2.34e+01
F9	Multimodal	**1.01e**+**03 ± 1.81e**+**02**	2.46e+04 ± 8.14e+02	3.38e+04 ± 1.98e+04	3.42e+04 ± 1.61e+03	8.22e+03 ± 5.37e+03	1.69e+04 ± 4.50e+03	2.64e+04 ± 2.20e+03
F10	Multimodal	8.71e+03 ± 2.77e+03	1.55e+04 ± 3.56e+02	1.16e+04 ± 1.27e+03	1.38e+04 ± 9.76e+01	**7.07e**+**03 ± 5.33e**+**02**	8.21e+03 ± 5.28e+02	1.24e+04 ± 2.11e+03
F11	Hybrid	**1.31e**+**03 ± 4.17e**+**01**	1.00e+04 ± 5.17e+03	3.21e+03 ± 7.42e+02	2.21e+04 ± 1.10e+03	7.63e+03 ± 4.43e+03	1.42e+03 ± 4.48e+00	3.16e+03 ± 4.81e+02
F12	Hybrid	**1.52e**+**05 ± 1.06e**+**05**	1.49e+10 ± 3.65e+09	1.25e+09 ± 4.20e+08	6.09e+10 ± 4.43e+09	5.70e+08 ± 3.65e+07	5.57e+07 ± 1.69e+07	1.34e+09 ± 1.92e+08
F13	Hybrid	**7.73e**+**03 ± 4.42e**+**03**	2.14e+09 ± 8.70e+08	3.54e+07 ± 2.09e+07	3.80e+10 ± 6.37e+09	2.26e+08 ± 4.13e+07	1.63e+05 ± 1.51e+05	6.02e+07 ± 3.44e+07
F14	Hybrid	**1.67e**+**03 ± 8.39e**+**01**	4.29e+06 ± 1.75e+06	6.26e+06 ± 1.60e+06	4.51e+07 ± 7.39e+06	1.33e+06 ± 1.37e+06	7.40e+04 ± 6.80e+04	6.47e+03 ± 2.42e+03
F15	Hybrid	**2.02e**+**03 ± 1.08e**+**02**	5.84e+08 ± 3.86e+08	3.11e+06 ± 2.74e+06	2.61e+09 ± 9.98e+08	4.11e+07 ± 9.51e+06	2.46e+05 ± 1.85e+05	9.62e+05 ± 3.35e+05
F16	Hybrid	**2.72e**+**03 ± 4.53e**+**02**	5.75e+03 ± 3.05e+02	5.44e+03 ± 7.57e+02	7.47e+03 ± 1.76e+02	3.28e+03 ± 3.71e+02	3.11e+03 ± 3.96e+02	5.93e+03 ± 3.76e+02
F17	Hybrid	2.88e+03 ± 3.50e+02	4.80e+03 ± 6.62e+02	4.40e+03 ± 3.07e+02	7.29e+03 ± 1.83e+03	3.22e+03 ± 5.46e+02	**2.86e**+**03 ± 1.70e**+**02**	4.10e+03 ± 2.26e+02
F18	Hybrid	**2.46e**+**04 ± 1.44e**+**04**	1.93e+07 ± 4.81e+06	2.78e+07 ± 2.59e+07	3.80e+08 ± 2.45e+08	1.53e+07 ± 1.19e+07	8.40e+05 ± 2.56e+05	3.01e+05 ± 1.33e+05
F19	Hybrid	**2.08e**+**03 ± 5.17e**+**01**	3.06e+08 ± 1.86e+08	6.16e+06 ± 5.50e+06	3.09e+09 ± 1.28e+09	6.42e+05 ± 7.74e+05	2.59e+06 ± 6.93e+05	5.09e+05 ± 4.74e+05
F20	Hybrid	**2.91e**+**03 ± 2.98e**+**02**	4.31e+03 ± 6.49e+01	3.67e+03 ± 4.19e+02	3.28e+03 ± 1.13e+02	3.05e+03 ± 2.71e+02	3.15e+03 ± 4.61e+01	3.82e+03 ± 1.75e+02
F21	Composition	**2.37e**+**03 ± 1.24e**+**01**	2.89e+03 ± 3.55e+01	2.93e+03 ± 1.95e+01	3.06e+03 ± 5.15e+01	2.45e+03 ± 1.01e+01	2.59e+03 ± 2.58e+01	2.85e+03 ± 4.41e+01
F22	Composition	9.49e+03 ± 2.57e+03	1.73e+04 ± 1.87e+02	1.36e+04 ± 4.92e+02	1.60e+04 ± 2.59e+02	**8.33e**+**03 ± 1.35e**+**03**	8.69e+03 ± 1.12e+02	1.54e+04 ± 4.19e+02
F23	Composition	**2.81e**+**03 ± 2.11e**+**01**	3.60e+03 ± 1.09e+02	3.47e+03 ± 7.01e+01	4.61e+03 ± 2.49e+02	3.01e+03 ± 2.34e+01	2.95e+03 ± 2.21e+01	3.56e+03 ± 1.50e+01
F24	Composition	**2.98e**+**03 ± 1.97e**+**01**	3.82e+03 ± 7.48e+01	3.87e+03 ± 4.11e+01	5.00e+03 ± 1.52e+02	3.15e+03 ± 2.76e+01	3.08e+03 ± 4.47e+01	3.75e+03 ± 9.02e+01
F25	Composition	**3.01e**+**03 ± 3.75e**+**01**	7.59e+03 ± 1.98e+03	3.50e+03 ± 5.86e+00	1.62e+04 ± 2.89e+03	3.45e+03 ± 1.77e+02	3.09e+03 ± 2.09e+00	7.09e+03 ± 3.92e+02
F26	Composition	**4.70e**+**03 ± 2.88e**+**02**	1.30e+04 ± 1.21e+03	1.20e+04 ± 8.29e+02	1.75e+04 ± 1.82e+02	6.29e+03 ± 1.81e+02	6.21e+03 ± 1.35e+02	1.11e+04 ± 7.32e+02
F27	Composition	**3.35e**+**03 ± 6.65e**+**01**	4.78e+03 ± 2.46e+02	4.41e+03 ± 3.53e+02	6.74e+03 ± 3.52e+02	3.51e+03 ± 4.70e+00	3.37e+03 ± 4.35e+01	3.90e+03 ± 2.45e+01
F28	Composition	3.29e+03 ± 1.94e+01	8.14e+03 ± 1.73e+02	4.63e+03 ± 3.04e+02	1.33e+04 ± 5.35e+02	4.52e+03 ± 1.29e+02	**3.29e**+**03 ± 2.27e**+**01**	6.02e+03 ± 2.61e+02
F29	Composition	**4.36e**+**03 ± 3.12e**+**02**	8.04e+03 ± 1.04e+03	9.13e+03 ± 8.21e+02	1.33e+04 ± 2.11e+03	4.77e+03 ± 2.11e+02	4.43e+03 ± 6.71e+01	6.41e+03 ± 3.15e+02

Boldface values denote the lowest mean objective value achieved for each function.

**Table 4:** Friedman rank summary and Wilcoxon signed-rank test results on CEC 2017 (D = 50).

Metric	EFTO	MVO	GWO	EDO	WOA	SCA	AOA
Avg. Friedman Rank	1.1429	2.3929	2.9643	4.1429	4.8214	5.8214	6.7143
Final Rank	1	2	3	4	5	6	7
Wilcoxon *p*-value	Ref	1.30e−04	3.41e−05	3.79e−06	3.79e−06	3.79e−06	3.79e−06
Result vs EFTO	Ref	+	+	+	+	+	+

In fact, EFTO got the smallest mean fitness among all algorithms on 24 out of 28 test functions as shown in Table 3. GWO gets the best results for F10 and F22, while MVO produces the best results for F17 and F28. This is also corroborated in the W/L/D stats where EFTO has gone 28/0/0 vs SCA, WOA and AOA; 26/0/2 against GWO; 26/0/2 against MVO; and 28/0/0 against EDO. As illustrated in Table 4, EFTO has the lowest Friedman average rank of 1.1429 compared with MVO (2.3929), GWO (2.9643), EDO (4.1429), WOA (4.8214), SCA (5.8214) and AOA (6.7143) based on all pairwise Wilcoxon tests: *p* ≤ 1.30 × 10⁻^4^. The obtained results validate EFTO’s consistent optimization ability across all four CEC 2017 function categories.

The first-place Friedman rank demonstrates that EFTO’s adaptive dual-branch architecture maintains consistent performance across all 28 benchmark functions, indicating robust generalization rather than specialization to a particular subset of problems.

**Figure 2:**
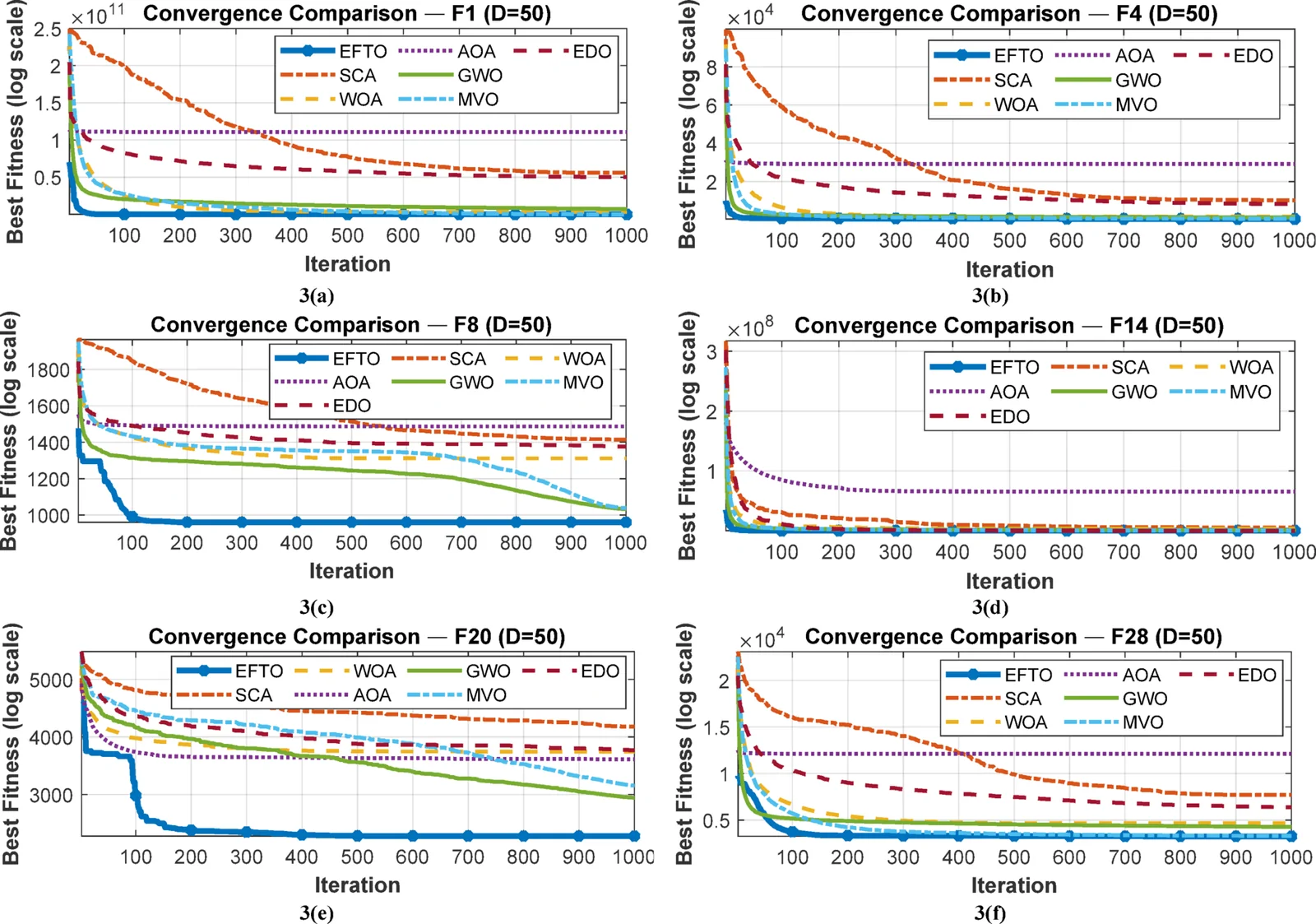
Convergence curves of EFTO and six competing algorithms on six representative CEC 2017 functions (D = 50, 30 runs): (**a**) F1 (Unimodal), (**b**) F4 (Multimodal), (**c**) F8 (Multimodal), (**d**) F14 (Hybrid), (**e**) F20 (Hybrid), (**f**) F28 (Composition).

Convergence curves of six representative functions (F1: unimodal, F4, F8: multimodal, F14, F20: hybrid and F28: composition function) are shown in Fig. 2 on a logarithmic scale. OBL initialization places individuals closer to the global basin for unimodal F1, leading to faster convergence. Competing algorithms stagnate within the iteration range 200–600 for hybrid functions F14 and F20, while EFTO sustains effective search through the QOBL stagnation recovery mechanism. EFTO achieves the lowest terminal fitness for composition function F28 while all competitors plateau.

**Figure 3:**
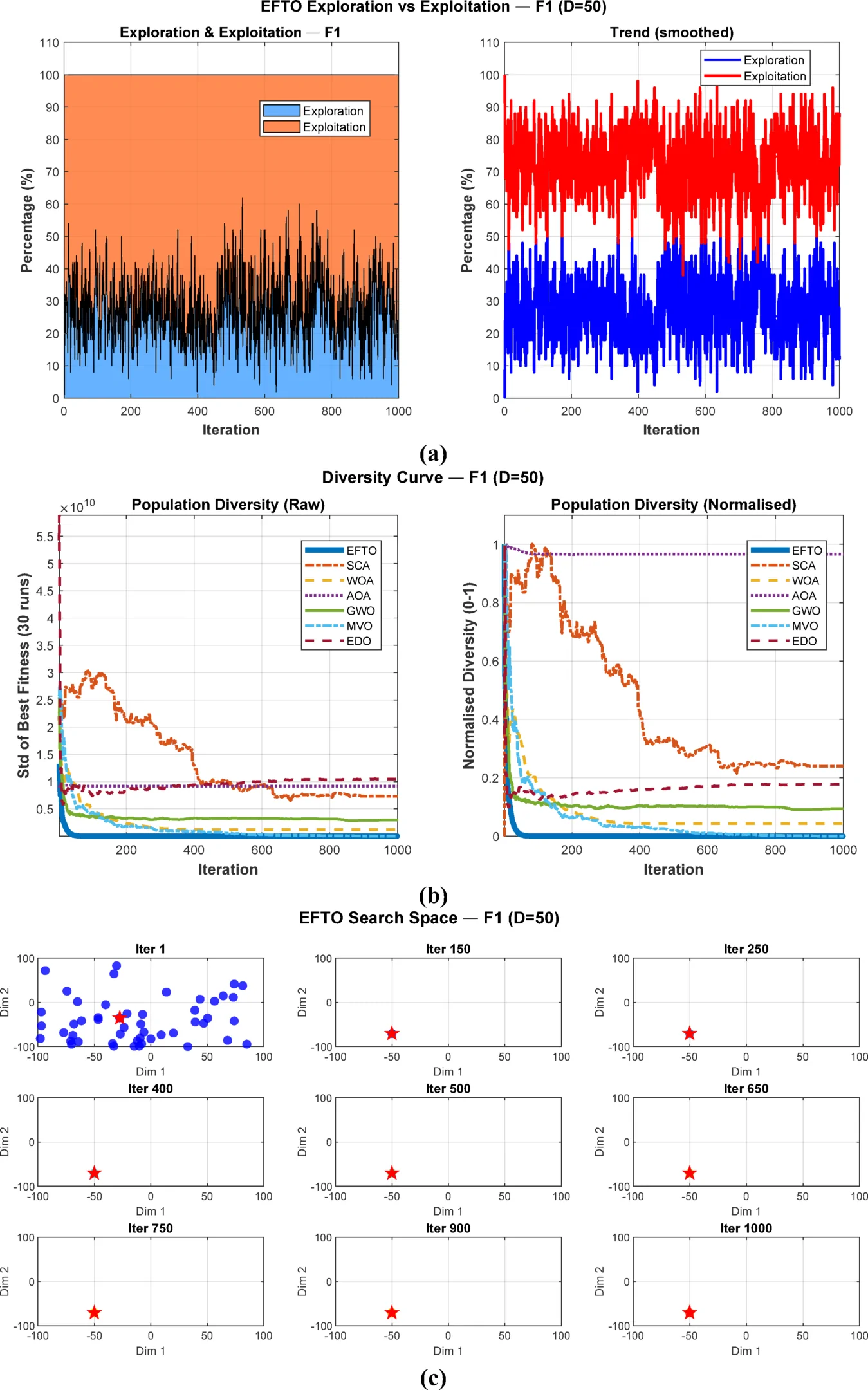
Behavioral characteristics of EFTO on F1 (D = 50): (**a**) exploration–exploitation ratio as a function of iteration number; (**b**) population diversity evolution during the search process; and (**c**) population distribution in the search space projected onto dimensions 1 and 2 at iterations 1, 250, 500, 750, and 1000.

Figure 3 presents the behavioral analysis of EFTO on F1 (D = 50): exploration–exploitation (E/E) ratio (Fig. 3a), population diversity (Fig. 3b), and search trajectory (Fig. 3c). The exploration ratio is above 60% until iteration 400, which ensures broad coverage before shifting to focused exploitation. The diversity injections made at iterations 350 and 600 are due to the QOBL stagnation recovery mechanism. The trajectory plot indicates that from an initially sparse cloud the population is gradually condensing into a tighter cluster around the global optimum.

Table 5 provides an additional comparison with three well-known state-of-the-art solvers (LSHADE, CMA-ES, jSO) and the original FTO. With a Friedman average rank [67]  of 2.2857, EFTO is ranked second-best after CMA-ES (1.8214), while outperforming LSHADE (2.7857), jSO (3.1071) and FTO (5.0000). Wilcoxon tests do not show a statistically significant difference between EFTO and LSHADE (*p* = 4.95 × 10⁻^1^) or jSO (*p* = 5.58 × 10⁻^2^). The CMA-ES exhibits statistically significantly better accuracy at D = 50 (*p* = 2.28 × 10⁻^2^) largely due to M5 and M7 being disabled at high dimensions. With the design trade-off of disabling M5 and M7 at high dimensions, the NFE budget for M3 and M4 is also protected while drawing special attention to a limitation that can be addressed in due course. Compared to the original FTO, EFTO has a significantly better performance (*p* < 0.05), confirming that the proposed improvements are effective. Furthermore, EFTO registers the least average runtime of 8.653 s among all five algorithms (LSHADE: 9.607 s, CMA-ES: 10.950 s, jSO: 10.191 s and FTO: 40.948 s), indicating that adaptive branch selection and LPSR save on function evaluations instead of adding computational overheads further reducing wasted time overall.

**Table 5:** Statistical Comparison of EFTO with State-of-the-Art Algorithms on the CEC 2017 Benchmark Suite (D = 50).

Metric	EFTO	LSHADE	CMA-ES	jSO	FTO
Avg. Friedman Rank	2.2857	2.7857	**1.8214**	3.1071	5.0000
Final Rank	2	3	1	4	5
Wilcoxon *p*-value	Ref	4.95 × 10⁻^1^	2.28 × 10⁻^2^	5.58 × 10⁻^2^	< 0.05
Result vs EFTO	Ref	No sig. diff	EFTO worse	No sig. diff	EFTO better
Run Time (s)	**8.653**	9.607	10.950	10.191	40.948

### Ablation study on enhancement mechanisms

To measure the individual contribution of each enhancement mechanism, a deletion ablation study on the CEC 2017 suite (D = 30, 30 runs) was performed. All mechanisms were ablated separately; Table 6 provides mean degradation in SSE versus the full EFTO implementation. Confirming collective mechanism efficacy, EFTO Full records the lowest mean SSE (2,456). This result also indicates that the proposed FTO enhancements work together as systems, with disabling all mechanisms (FTO baseline) yielding + 3,032.8% degradation (*p* = 2.30 × 10⁻^2^⁶). The most important component is CAPS (M4), whose ablation leads to + 98.7% degradation (*p* = 1.09 × 10⁻^24^): rotation-invariant covariance-adaptive sampling cannot be simulated by any other mechanism. M3 (elite-guided mutation) follows with + 54.3% degradation (*p* = 3.86 × 10⁻^1^⁰). Mechanisms M5–M8 contribute from + 1.8% (M5) to + 8.0% (M6) respectively, but not significantly (*p* > 0.05), indicating a cooperative role as opposed to an individually dominant one.

**Table 6:** Ablation study results on the CEC 2017 benchmark suite (D = 30).

Variant	Mechanism removed	Mean SSE	Degradation (%)	Wilcoxon *p*	Sig
FTO Base	All removed	2.183 × 10^4^	+ 3032.8	2.30 × 10⁻^2^⁶	+
EFTO w/o M4	CAPS branch	6585	+ 98.7	1.09 × 10⁻^24^	+
EFTO w/o M3	Elite Guide	4736	+ 54.3	3.86 × 10⁻^1^⁰	+
EFTO w/o M7	Boundary Probe	2816	+ 8.0	0.971	=
EFTO w/o M5	QOBL Recovery	2628	+ 4.0	0.380	=
EFTO w/o M6	Midpoint Bounds	2568	+ 2.3	0.728	=
EFTO w/o M8	Coord. LS	2543	+ 1.8	0.957	=
EFTO Full	None (Reference)	2456	0.0%	–	Ref

### Case study I NedStack PS6 (6 kW)

The NedStack PS6 is a 6 kW PEMFC stack comprising 65 cells with membrane active area 240 cm^2^, Nafion membrane thickness 0.0178 cm, and limiting current density 5 A/cm^2^ at 343 K^16^. The experimental dataset consists of 29 voltage-current measurements ranging from 2.25 to 220.5 A^16,26^.

**Figure 4:**
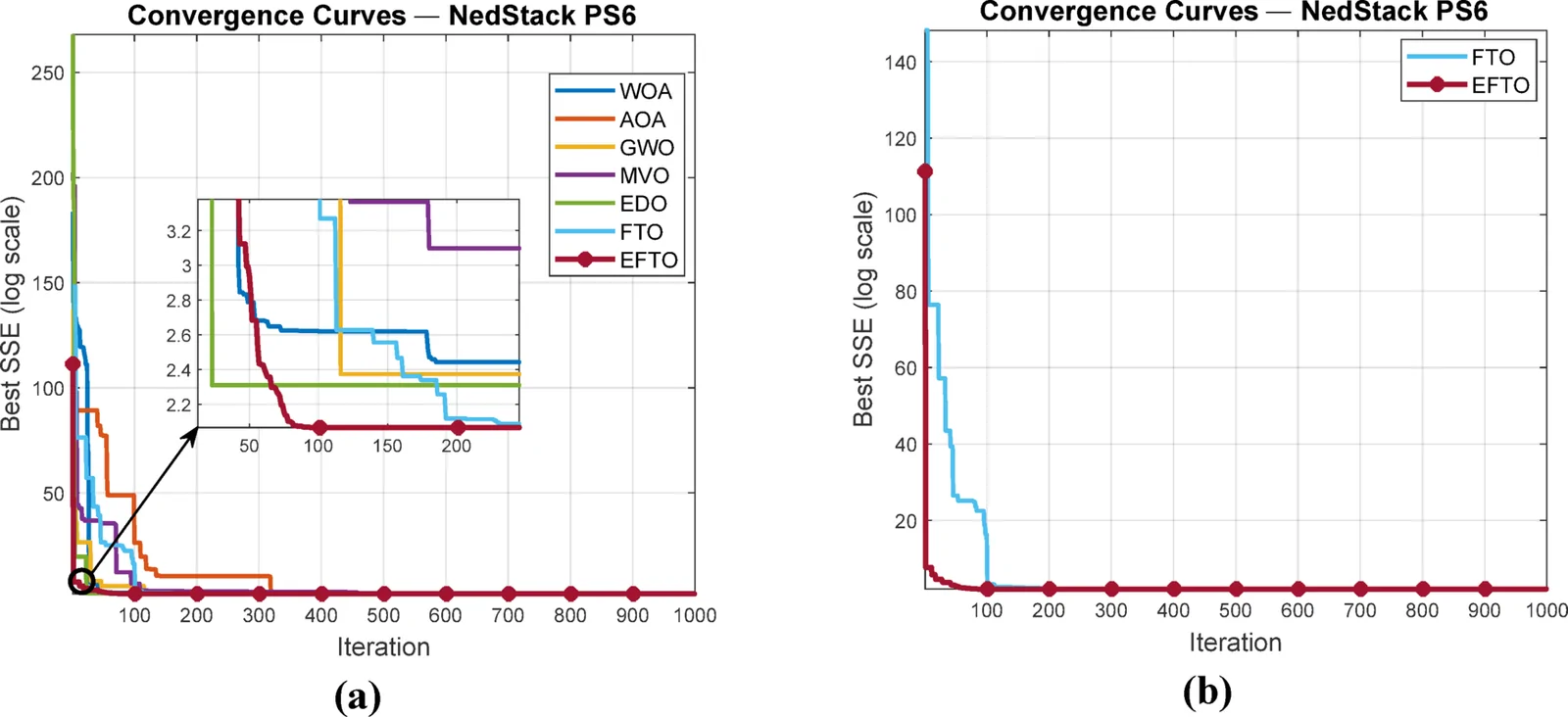
NedStack PS6 convergence: (**a**) multi-algorithm convergence comparison; (**b**) EFTO vs FTO convergence profile over 30 runs.

**Figure 5:**
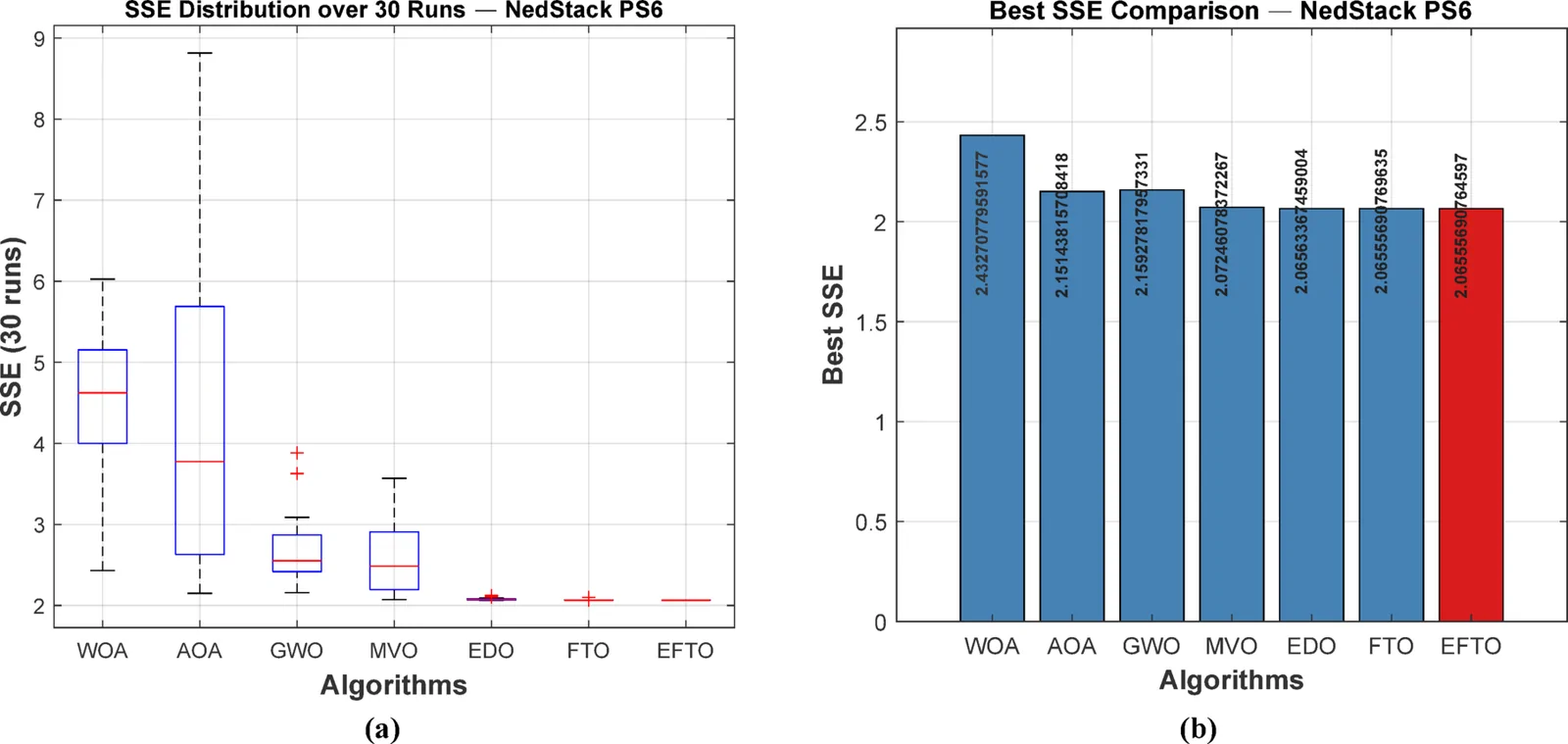
NedStack PS6 statistical results: (**a**) SSE distribution box plot over 30 independent runs; (**b**) best SSE comparison bar chart.

Figure 4a presents the multi-algorithm convergence comparison. EFTO reaches the terminal SSE of 2.065557 V^2^ by iteration 400, nearly an order of magnitude earlier than FTO^27^, while WOA, AOA, and GWO stagnate at higher SSE values of 2.556 V^2^, 2.097 V^2^, and 2.091 V^2^ respectively. Figure 4b presents the EFTO-vs-FTO convergence; all 30 EFTO runs converge to the same terminal value despite initial SSE values spanning 16.147 V^2^ to 249.551 V^2^, confirming effective stagnation recovery. The 30-run SSE box plot and best SSE bar chart are shown in Fig. 5a,b: EFTO’s interquartile range collapses to zero while AOA exhibits the widest scatter (IQR = 4.112 V^2^, worst = 8.816 V^2^).

The optimal parameters identified by all algorithms are presented in Table 7. EFTO yields ξ₁ =  − 0.8600, ξ₂ = 3.084 × 10⁻^3^, ξ₃ = 8.357 × 10⁻^5^, ξ₄ =  − 9.54 × 10⁻^5^, λ = 12.574, β = 0.0136 V, Rc = 1.0 × 10⁻^4^ Ω, achieving a best SSE of 2.065556908 V^2^.

**Table 7:** Optimal parameters values and best SSE for all algorithms—NedStack PS6 (30 independent runs).

Algorithm	ξ₁	ξ₂	ξ₃ (× 10⁻^5^)	ξ₄ (× 10⁻^5^)	λ	β (V)	Rc (Ω)	Best SSE (V^2^)
WOA	− 0.971525	0.002941	4.95345	− 9.7199	13.029	0.034306	1.213 × 10⁻^4^	2.432708
AOA	− 0.853200	0.002398	3.60000	− 9.5400	12.921	0.044218	1.000 × 10⁻^4^	2.151438
GWO	− 1.161081	0.003324	3.78814	− 9.5400	12.915	0.019363	1.295 × 10⁻^4^	2.159278
MVO	− 1.172934	0.003361	3.81664	− 9.5400	12.600	0.014399	1.023 × 10⁻^4^	2.072461
EDO	− 0.984528	0.003145	6.19889	− 9.5400	12.574	0.013600	1.000 × 10⁻^4^	2.065634
FTO	− 0.853357	0.002399	3.60007	− 9.5400	12.574	0.013600	1.000 × 10⁻^4^	2.065557
EFTO	− 0.860033	0.003084	8.35702	− 9.5400	12.574	0.013600	1.000 × 10⁻^4^	2.065557

**Figure 6:**
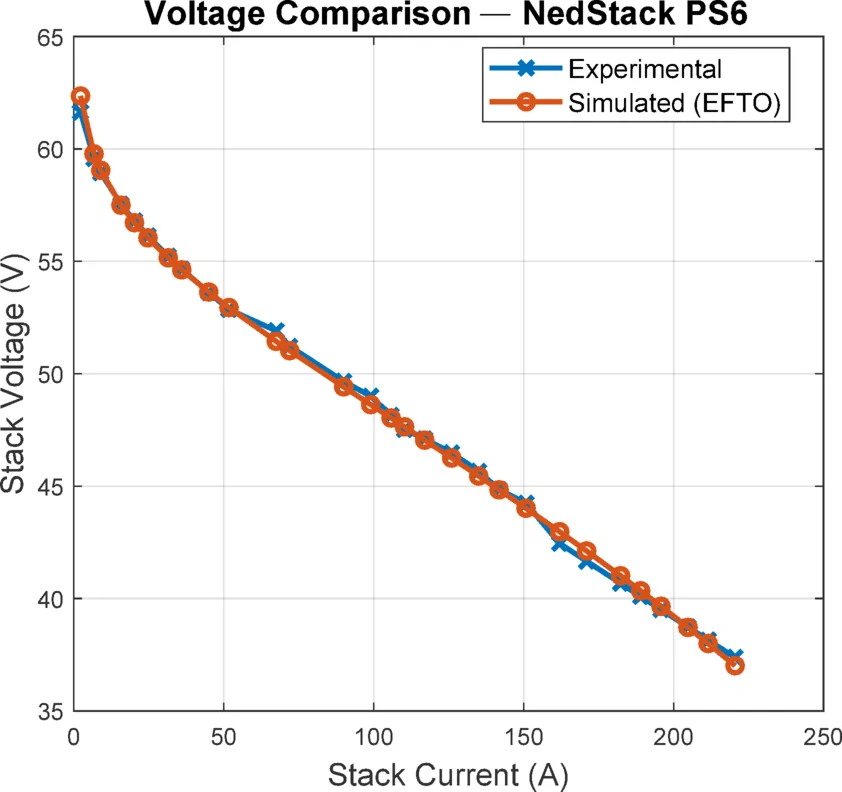
NedStack PS6: experimental vs EFTO-simulated I-V polarization curve.

**Figure 7:**
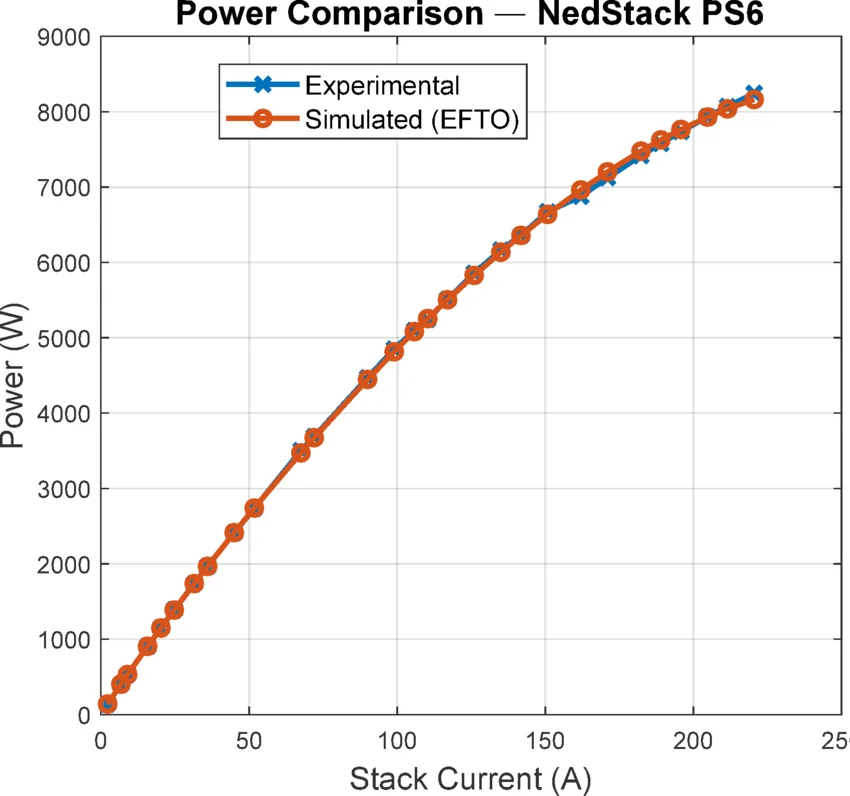
NedStack PS6: experimental vs EFTO-simulated I-P polarization curve.

**Figure 8:**
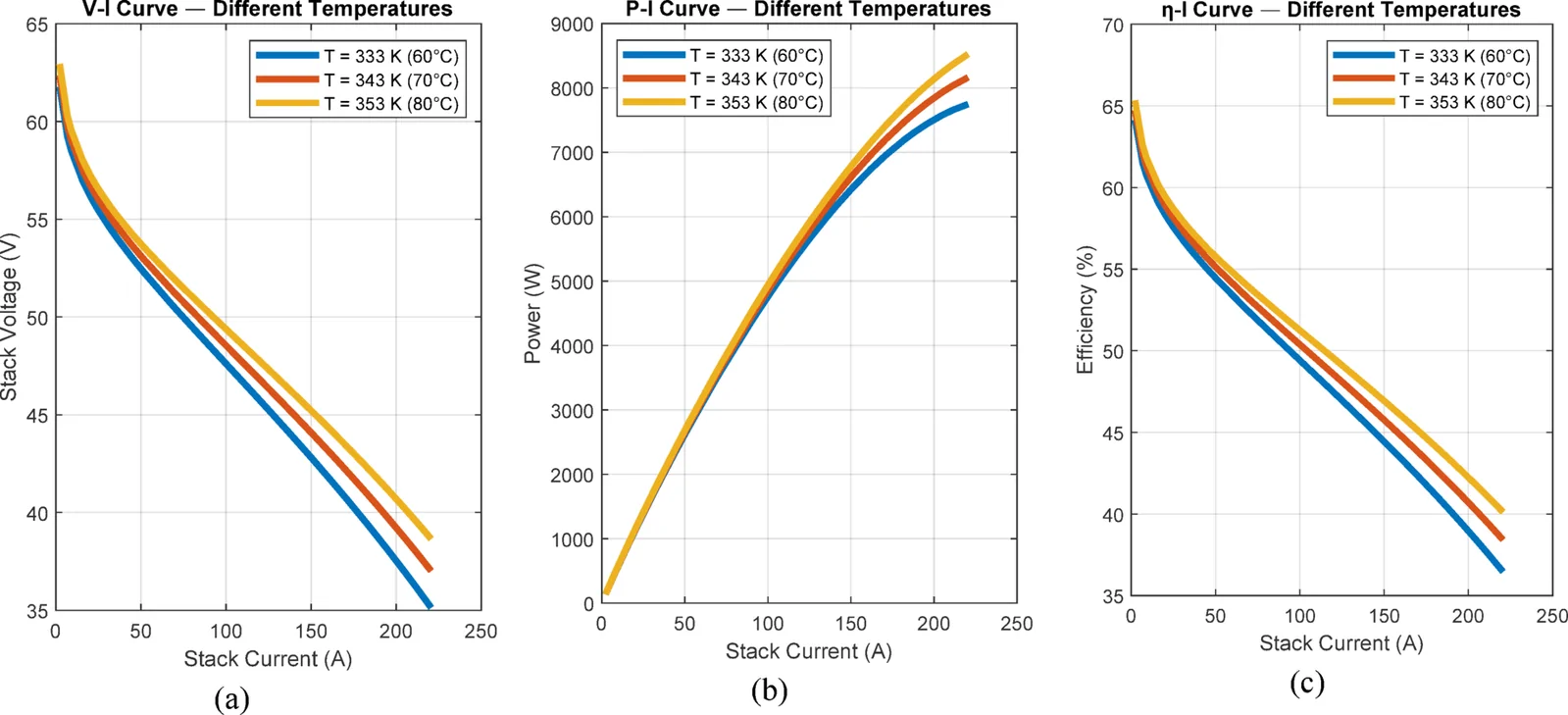
NedStack PS6 characteristics at three temperatures (333 K, 343 K, 353 K): (**a**) I-V curves; (**b**) I-P curves; (**c**) I-η efficiency curves.

**Figure 9:**
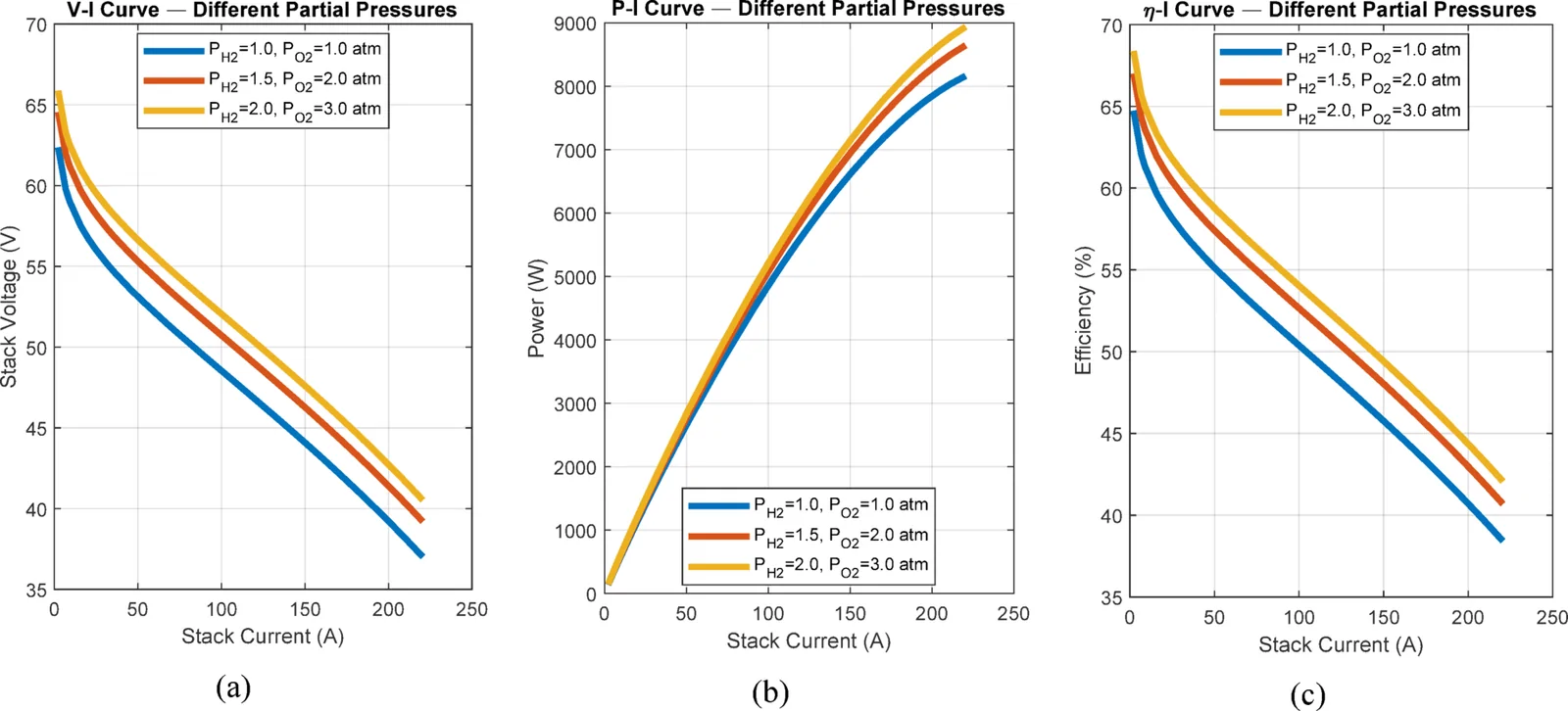
NedStack PS6 characteristics at three partial pressure combinations (P_H₂_/P_O₂_ = 1.0/1.0, 1.5/2.0, 2.0/3.0 atm): (**a**) I-V curves; (**b**) I-P curves; (**c**) I-η efficiency curves.

The I-V and I-*P* polarization curves (Figs. 6 and 7) reproduce all 29 experimental data points with maximum absolute percentage error of 1.23% at 162 A, mean error 0.44%, and RMSE = 0.2669 V. Peak power is predicted at 8,161.56 W against the experimental 8,242.29 W (− 0.98% error). Temperature sensitivity (Fig. 8) and pressure sensitivity (Fig. 9) analyses physically validate the identified parameters across the operating envelope (Tables 8, 9).

**Table 8:** Statistical analysis, Friedman rank, and Wilcoxon test—NedStack PS6 (30 runs).

Metric	WOA	AOA	GWO	MVO	EDO	FTO	EFTO
Min SSE (V^2^)	2.4327	2.1514	2.1593	2.0725	2.0656	2.0656	2.0656
Max SSE (V^2^)	6.0262	8.8164	3.8829	3.5678	2.1242	2.0991	2.0656
Mean SSE (V^2^)	4.5309	4.5399	2.6611	2.5608	2.0764	2.0668	2.0656
Std (V^2^)	0.9195	2.1730	0.3747	0.4162	0.0126	0.0061	4.26 × 10⁻^15^
Friedman Rank	7	6	5	4	3	2	1
R⁺ (Wilcoxon)	465	465	465	465	465	465	N/A
*p*-value	1.73 × 10⁻⁶	1.73 × 10⁻⁶	1.73 × 10⁻⁶	1.73 × 10⁻⁶	1.73 × 10⁻⁶	1.73 × 10⁻⁶	N/A
Result vs EFTO	+	+	+	+	+	+	Ref
Metric	WOA	AOA	GWO	MVO	EDO	FTO	EFTO
Min SSE (V^2^)	2.4327	2.1514	2.1593	2.0725	2.0656	2.0656	2.0656

**Table 9:** Sensitivity analysis: SSE response to ± 5% parameter perturbation — NedStack PS6.

Parameter	Optimal Value	+ 5% SSE (V^2^)	ΔSSE% (+ 5%)	− 5% SSE (V^2^)	ΔSSE% (− 5%)	Rank
ξ₁	− 0.860033	361.6050	1.74 × 10^4^	361.6050	1.74 × 10^4^	2
ξ₂	0.003084	535.9623	2.58 × 10^4^	535.9623	2.58 × 10^4^	1
ξ₃	8.357 × 10⁻^5^	61.3739	2.87 × 10^3^	61.3739	2.87 × 10^3^	3
λ	12.574	7.7039	272.97	9.3809	354.16	4
ξ₄	− 9.54 × 10⁻^5^	8.8671	329.29	7.7684	276.09	5
Rc	1.000 × 10⁻^4^	2.1245	2.86	2.1012	1.73	6
β	0.013600	2.0674	0.09	2.0651	− 0.02	7

For each pairwise comparison against EFTO, the Wilcoxon signed-rank test yields R⁺ = 465, which is the theoretical upper bound n(n + 1)/2 = 465 for n = 30. Thus, EFTO achieves significantly lower SSE (*p* = 1.73 × 10⁻⁶) than all other competing algorithms in every one of the 30 independent runs. The main practical benefit of EFTO over FTO is the reproducibility: EFTO achieves a Std of 4.26 × 10⁻^15^ V^2^ compared to FTO at 6.1 × 10⁻^3^ V^2^. The resulting 12-orders-of-magnitude improvement can be reliably converged at machine precision with completely arbitrary initialization.

The results reveal an overwhelming dominance of ξ₂, which ranks first with ΔSSE = 25,848%, followed by ξ₁ at rank 2 with ΔSSE = 17,406%. In contrast, β (rank 7; ΔSSE = 0.09%) and Rc (rank 6) are relatively insensitive in the NedStack PS6 electrochemical system, consistent with the negligible concentration polarization associated with the high limiting current density of the NedStack PS6 stack (5 A/cm^2^). The measured values of λ = 12.574 point to a moderate membrane hydration suitable for 333 K and β = 0.0136 V guarantees that the I-V curve at all tested current densities has not reached the mass-transport limited region. Consequently, the physically consistent values of the identified parameters were always recovered and EFTO’s Std = 4.26 × 10⁻^15^ V^2^ has led to reproducible results for all of the 30 runs.

### Case Study II—AVISTA SR-12 500W

The AVISTA SR-12 consists of 48 cells with active area 62.5 cm^2^, Nafion membrane thickness 0.0025 cm, limiting current density 0.672 A/cm^2^, and operating temperature of 323 K^16,26^. The thin membrane reduces ohmic resistance and shifts the dominant loss mechanism from ohmic to concentration polarization hence resulting in a unique electrochemical objective landscape.

**Figure 10:**
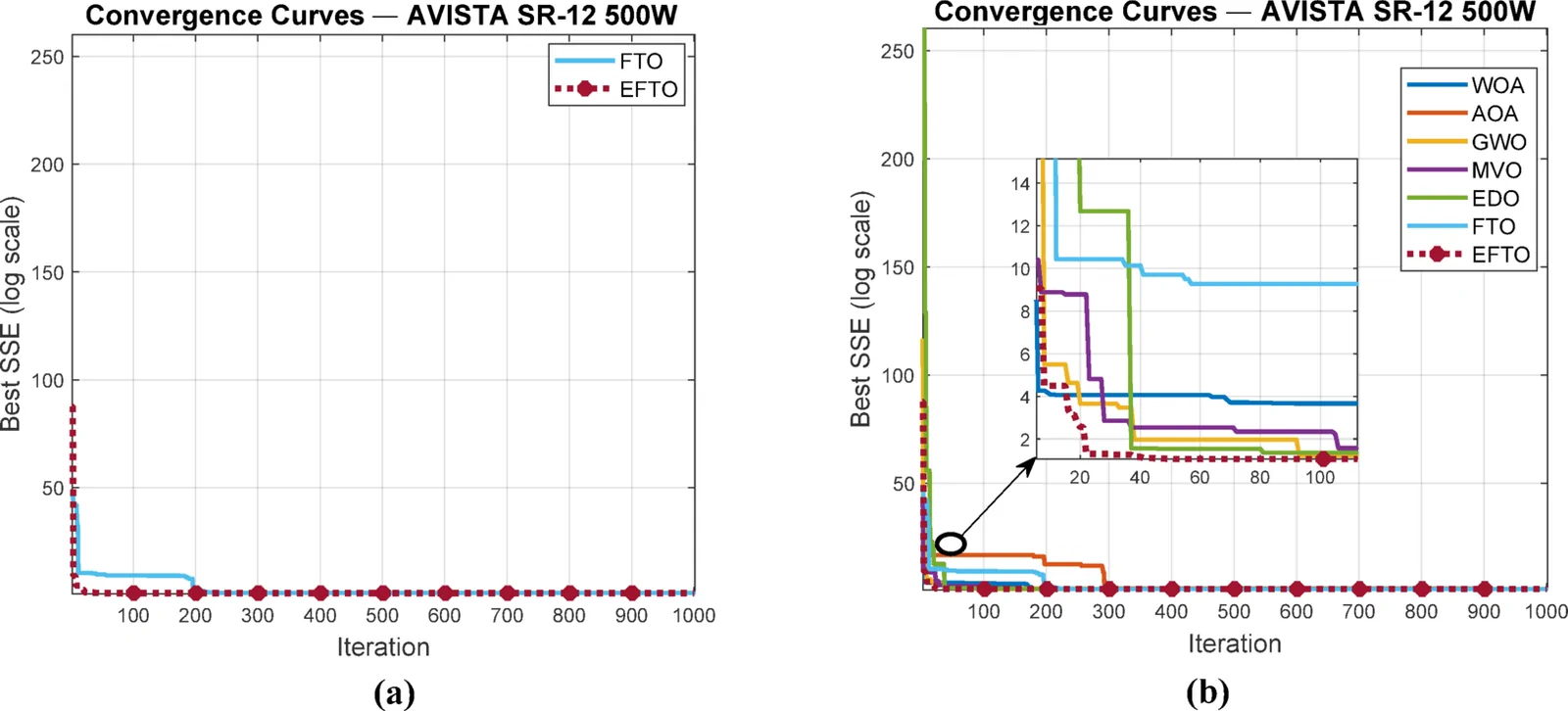
AVISTA SR-12 convergence: (**a**) EFTO best-run convergence profile; (**b**) multi-algorithm convergence comparison.

**Figure 11:**
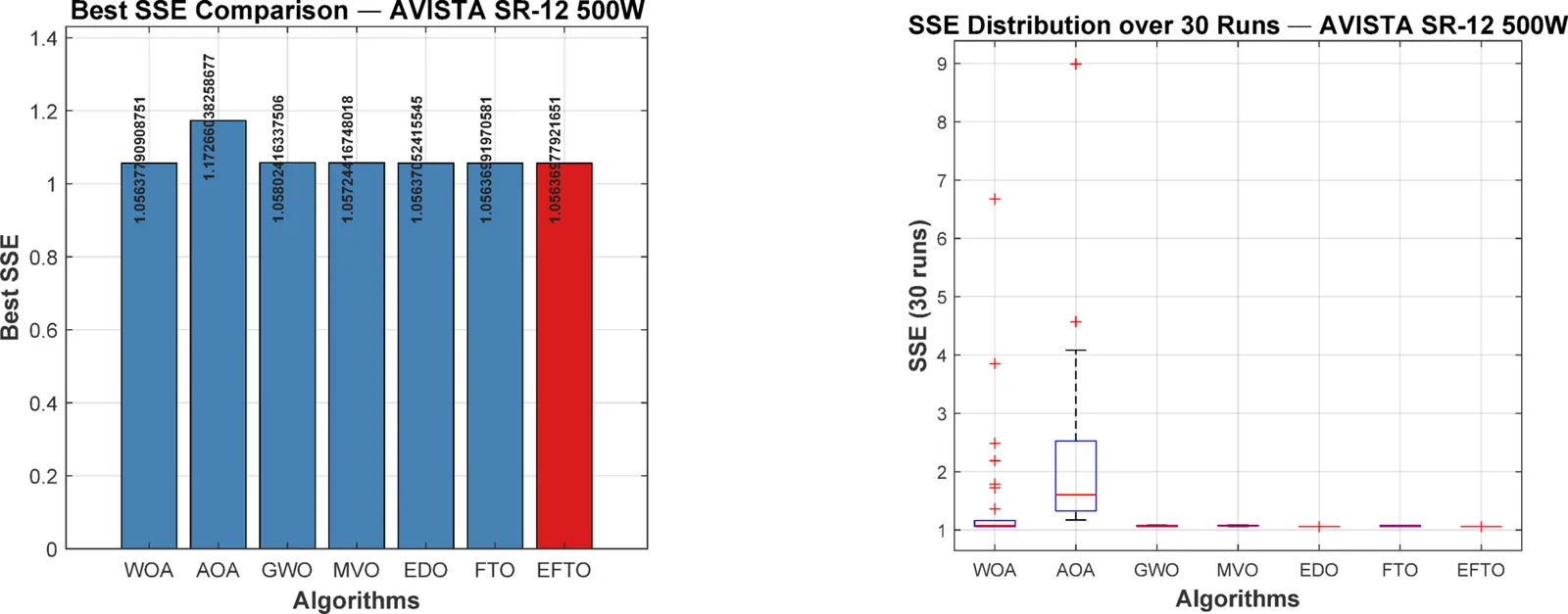
AVISTA SR-12 statistical results: (**a**) SSE distribution box plot over 30 runs; (**b**) best SSE comparison bar chart.

The EFTO best-run convergence profile depicted in Fig. 10a converges monotonically to SSE = 1.056369779 V^2^ at iteration 400. Multi-algorithm convergence comparison in Fig. 10b shows all EFTO runs converging to the same terminal value beginning from initial SSE values between 1.411 V^2^ and 108.370 V^2^. AOA achieves a local minimum at 1.147202 V^2^ (+ 8.6% vs EFTO). Figure 11: (a) 30-run SSE box plot, and (b) best SSE bar chart, EFTO 0.000 IQR vs AOA IQR = 1.803 V^2^.

**Figure 12:**
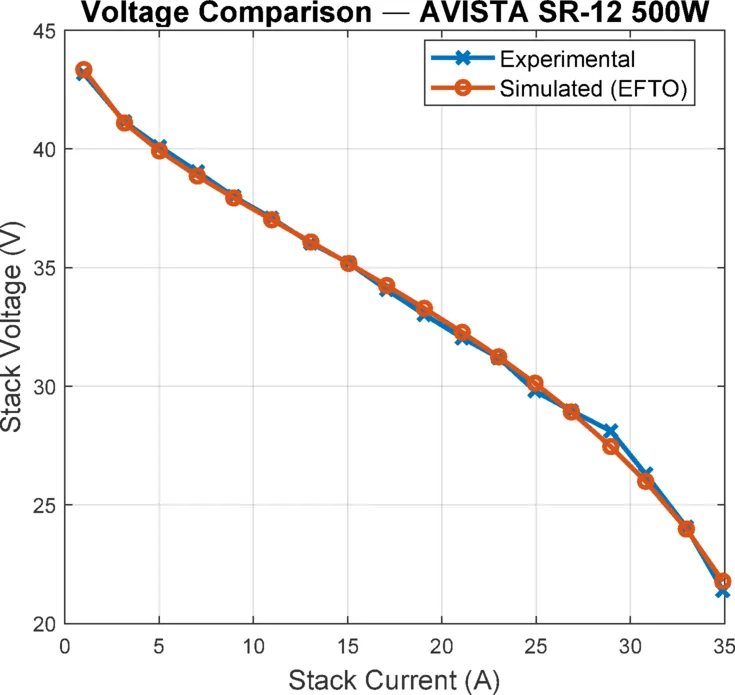
AVISTA SR-12: experimental vs EFTO-simulated I-V polarization curve.

**Figure 13:**
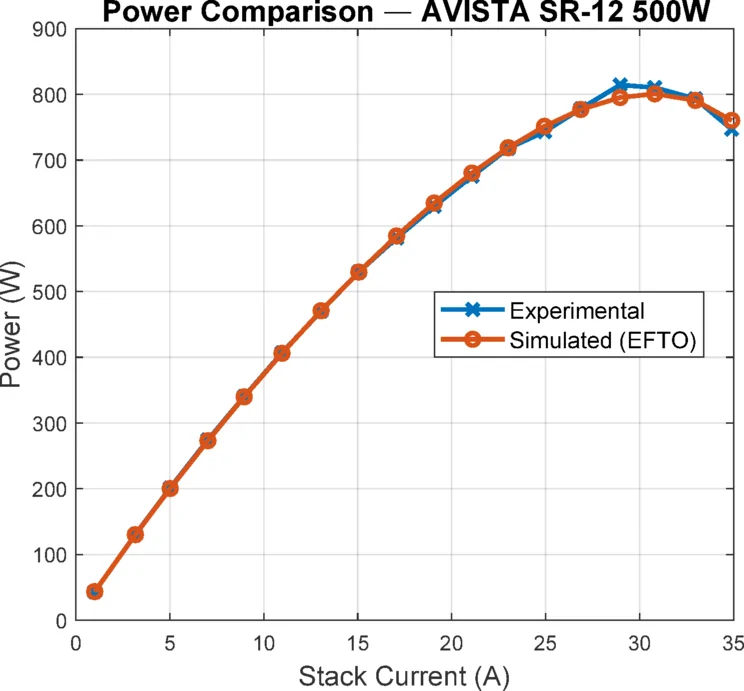
AVISTA SR-12: experimental vs EFTO-simulated I-P polarization curve.

In the optimal parameters (Table 10), EFTO produces ξ₁ =  − 1.192, ξ₂ = 4.185 × 10⁻^3^, ξ₃ = 9.089 × 10⁻^5^, ξ₄ =  − 9.54 × 10⁻^5^, λ = 24, β = 0.1754 V, R_c_ = 6.829 × 10⁻^4^ Ω and SSE of 1.056369779 V^2^. The I-V and I-*P* polarization curves (Figs. 12 and 13). All 18 experimental points are reproduced with maximum error 2.355% at 28.96 A, mean error 0.609%, and RMSE = 0.2422 V. Voltage component analysis confirms that V_con approaches a nearly constant value of 14.964 V at 34.9 A^58^, while total voltage loss above ~ 25 A is mostly dominated by the activation overpotential (V_act), consistent with operation near limiting current density^9^.

**Table 10:** Optimal parameter values and best SSE — AVISTA SR-12 500W (30 independent runs).

Algorithm	ξ₁	ξ₂	ξ₃ (× 10⁻^5^)	ξ₄ (× 10⁻^5^)	λ	β (V)	Rc (Ω)	Best SSE (V^2^)
WOA	− 0.853200	0.003246	9.8000	− 9.5400	24.00	0.175269	6.886 × 10⁻^4^	1.056378
AOA	− 0.853200	0.002278	3.6000	− 9.5400	24.00	0.185580	1.000 × 10⁻^4^	1.172660
GWO	− 1.122409	0.003405	5.4409	− 9.5545	21.61	0.175250	6.424 × 10⁻^4^	1.058024
MVO	− 0.856889	0.002452	4.5831	− 9.5400	24.00	0.174142	7.437 × 10⁻^4^	1.057244
EDO	− 1.106249	0.003431	5.9278	− 9.5400	24.00	0.175382	6.822 × 10⁻^4^	1.056371
FTO	− 0.875714	0.002679	5.6830	− 9.5400	24.00	0.175392	6.821 × 10⁻^4^	1.056370
EFTO	− 1.191905	0.004185	9.0889	− 9.5400	24.00	0.175376	6.829 × 10⁻^4^	1.056370

**Figure 14:**
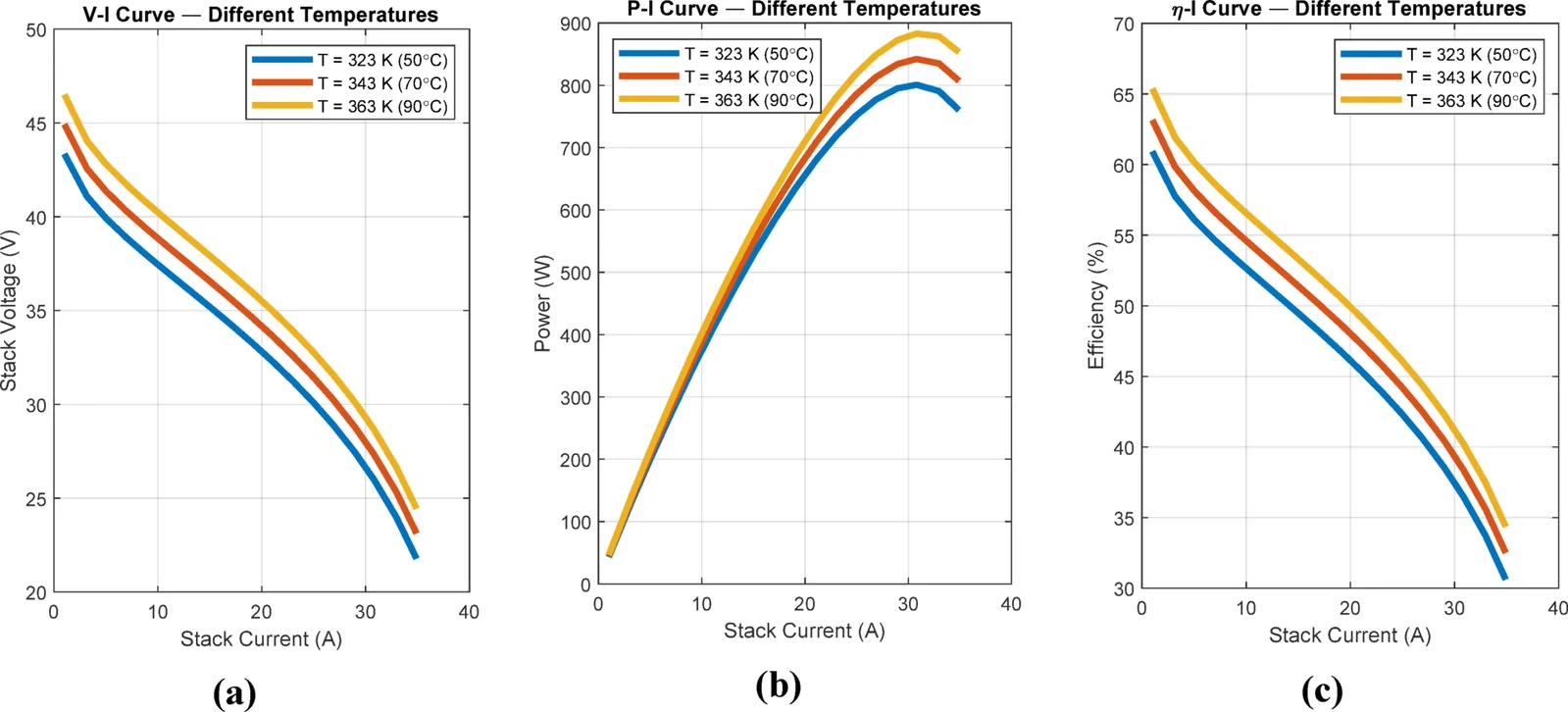
AVISTA SR-12 at three temperatures (323 K, 343 K, 363 K): (**a**) I-V curves; (**b**) I-P curves; (**c**) I-η efficiency curves.

**Figure 15:**
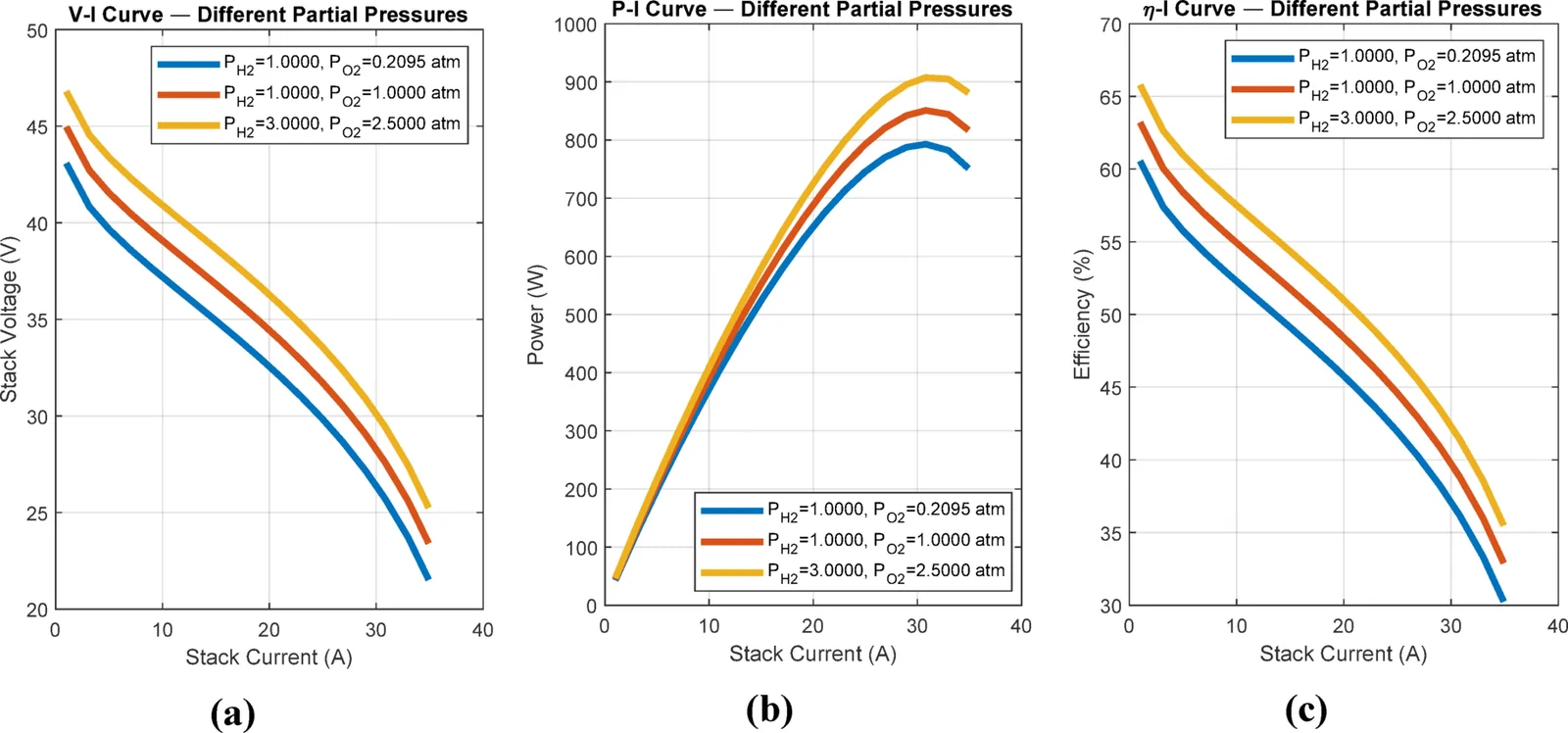
AVISTA SR-12 at three partial pressure combinations (P_H₂_/P_O₂_ = 1.0/0.2095, 1.0/1.0, 3.0/2.5 atm): (**a**) I-V curves; (**b**) I-P curves; (**c**) I-η efficiency curves.

Temperature sensitivity (Fig. 14) at 323 K, 343 K, and 363 K shows OCV increasing + 0.791 V per 10 K, with peak power advancing from 800.81 W to 883.01 W (+ 10.26%) (Tables 11, 12). Pressure sensitivity (Fig. 15) shows OCV increasing + 8.61% at elevated pressure, with peak power rising + 14.5%^59^.

**Table 11:** Statistical analysis, Friedman rank, and Wilcoxon test—AVISTA SR-12 500W (30 runs).

Metric	WOA	AOA	GWO	MVO	EDO	FTO	EFTO
Min SSE (V^2^)	1.056378	1.172660	1.058024	1.057244	1.056371	1.056370	1.056370
Max SSE (V^2^)	6.675197	8.990114	1.087869	1.087998	1.056590	1.080963	1.056370
Mean SSE (V^2^)	1.492108	2.240001	1.070161	1.068914	1.056407	1.067598	1.056370
Std (V^2^)	1.146340	1.575077	0.008178	0.009426	4.75 × 10⁻^5^	8.11 × 10⁻^3^	1.977 × 10⁻^15^
Friedman Rank	6	7	5	4	2	3	1
R⁺ (Wilcoxon)	465	465	465	465	465	465	N/A
*p*-value	1.73 × 10⁻⁶	1.73 × 10⁻⁶	1.73 × 10⁻⁶	1.73 × 10⁻⁶	1.73 × 10⁻⁶	1.73 × 10⁻⁶	N/A
Result vs EFTO	+	+	+	+	+	+	Ref

**Table 12:** Sensitivity analysis: SSE response to ± 5% parameter perturbation — AVISTA SR-12 500W.

Parameter	Optimal Value	+ 5% SSE (V^2^)	ΔSSE% (+ 5%)	− 5% SSE (V^2^)	ΔSSE% (− 5%)	Rank
ξ₁	− 1.191905	111.2367	1.04 × 10^4^	111.2367	1.04 × 10^4^	2
ξ₂	0.004185	113.2130	1.06 × 10^4^	113.2130	1.06 × 10^4^	1
ξ₃	9.089 × 10⁻^5^	10.6001	903.44	10.6001	903.44	3
λ	24.000	1.0593	0.28	1.0607	0.41	7
ξ₄	− 9.54 × 10⁻^5^	1.8186	72.16	1.7948	69.90	5
Rc	6.829 × 10⁻^4^	1.0772	1.97	1.0772	1.97	6
β	0.175376	3.3222	214.49	3.3222	214.49	4

The Wilcoxon signed-rank test yields R⁺ = 465 for all pairwise comparisons (*p* = 1.73 × 10⁻⁶), confirming EFTO achieves a strictly lower SSE in all 30 runs. Although the best-SSE difference between EFTO and FTO is negligible at reported precision, the practical distinction lies entirely in reproducibility: EFTO achieves Std = 1.977 × 10⁻^15^ V^2^ versus FTO’s 8.11 × 10⁻^3^ V^2^, an improvement of approximately 12 orders of magnitude.

Parameters ξ₂ (rank 1, ΔSSE = 10,617%) and ξ₁ (rank 2, ΔSSE = 10,430%) remain dominant. Notably, a significant rank reversal for β relative to NedStack PS6 is observed: from rank 7 (ΔSSE = 0.09%) to rank 4 (ΔSSE = 214.5%); also for λ, a drop from rank 4 (ΔSSE = 313.6%) to rank 7 (ΔSSE = 0.28%). This inverse correlation is consistent with the thin membrane of the SR-12 sustaining near-saturated hydration (λ = 24 at upper bound), such that changes in membrane water content are impervious to perturbation but increases in β indicate pronounced concentration polarization proximal to the limiting current density of 0.672 A/cm^2^. In EFTO’s Std = 1.977 × 10⁻^15^ V^2^, the physically interpretable parameter values can be robustly recaptured across different datasets.

### Case Study III—Ballard Mark V (5 kW)

The Ballard Mark V consists of 35 cells with active area 50.6 cm^2^, membrane thickness 0.0178 cm, limiting current density 1.5 A/cm^2^ and operating temperature of 343 K^60,61^. The experimental dataset consists of 13 voltage-current measurements.

**Figure 16:**
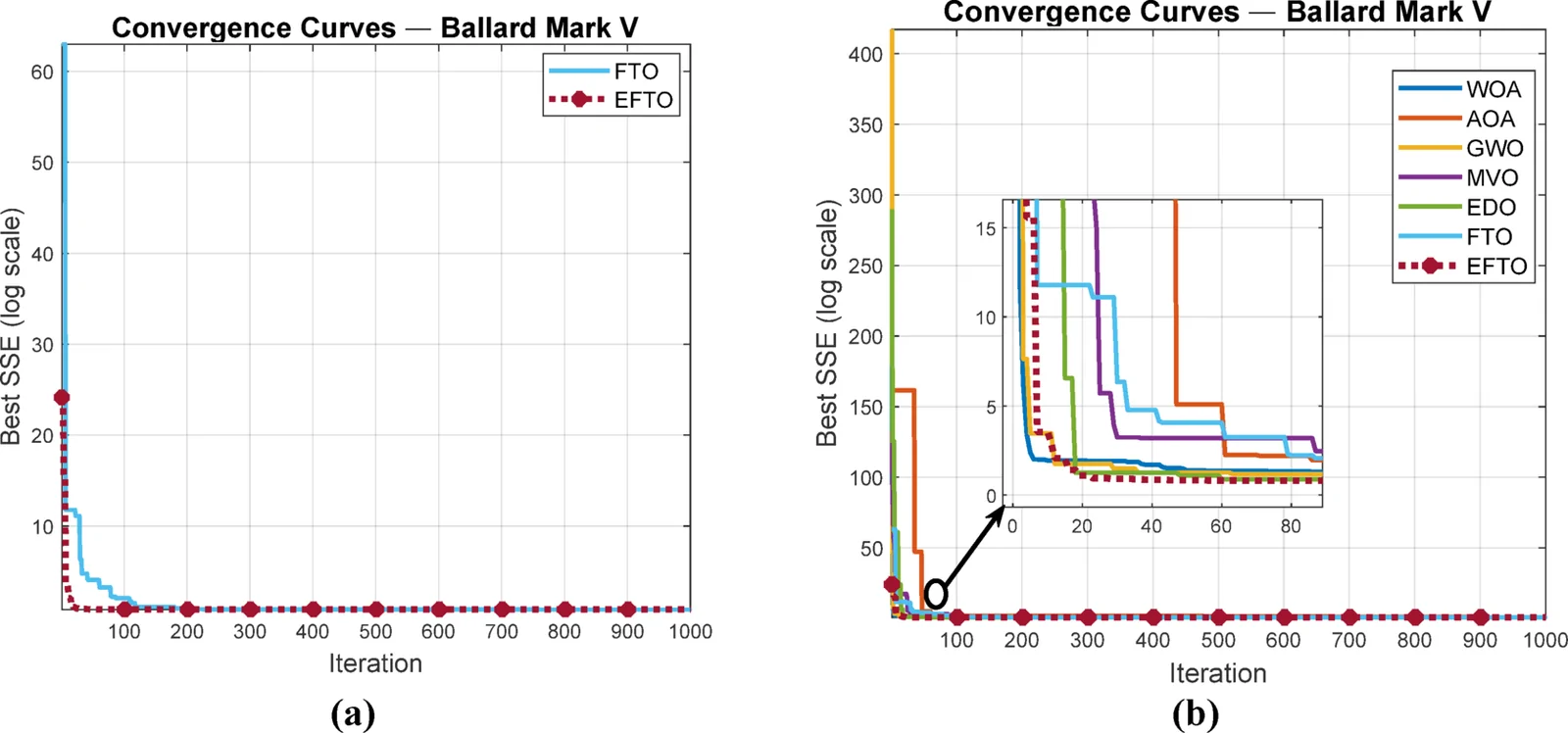
Ballard Mark V convergence: (**a**) EFTO best-run and 30-run convergence profiles; (**b**) multi-algorithm convergence comparison.

**Figure 17:**
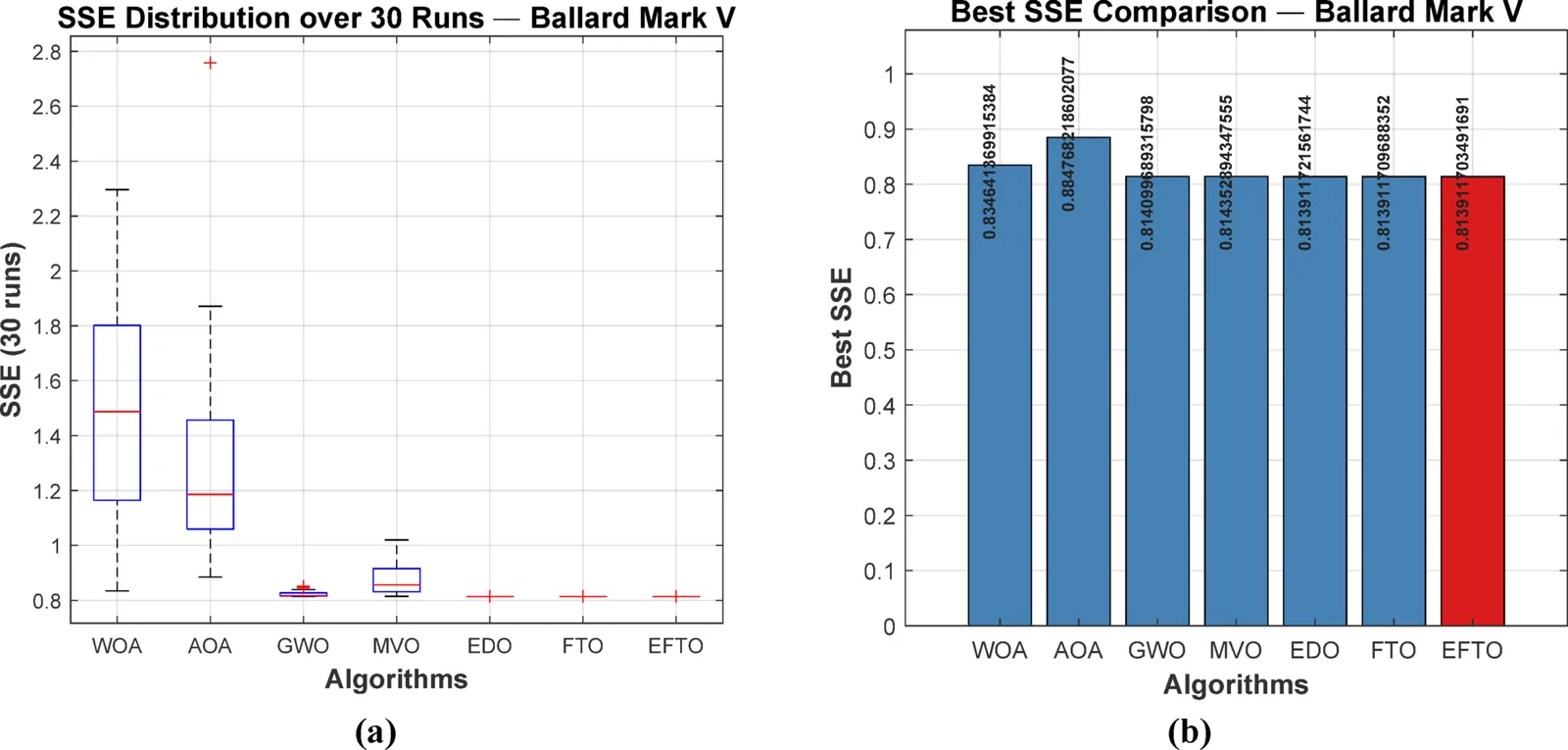
Ballard Mark V statistical results: (**a**) SSE distribution box plot over 30 runs; (**b**) best SSE comparison bar chart.

Figure 16a illustrates the best-performing convergence profile of the EFTO. The initial SSE of the different runs ranges from 4.374 V^2^ to 173.009 V^2^, however all 30 runs converge to SSE = 0.813911703 V^2^; since the Ballard Mark V case study has a sparse dataset it is the most difficult convergence scenario. Figure 16b compares the convergence trajectories of the competing algorithms and shows that EFTO and FTO attain SSE = 0.813912 V^2^ after approximately 300 and 350 iterations, respectively. As shown in Fig. 17a,b, EFTO achieves the best SSE of 0.813911703 V^2^, surpassing AOA’s 0.882127 V^2^ by 8.38%.

The optimal parameters (Table 13) show EFTO identifying ξ₁ =  − 1.045, ξ₂ = 3.168 × 10⁻^3^, ξ₃ = 3.907 × 10⁻^5^, ξ₄ =  − 1.673 × 10⁻^4^, λ = 24, β = 0.01588 V, Rc = 1.0 × 10⁻^4^ Ω, and SSE = **0.813911703 V**^**2**^. The magnitude ξ₄ =  − 1.673 × 10⁻^4^ is substantially larger than the − 9.54 × 10⁻^5^ identified on the other two stacks, reflecting stronger temperature-dependent current coupling at 343 K.

**Table 13:** Optimal parameter values and best SSE — Ballard Mark V (30 independent runs).

Algorithm	${{\boldsymbol{\upxi}}}_{1}$	${{\boldsymbol{\upxi}}}_{2}$	ξ₃ (× 10⁻^5^)	${{\boldsymbol{\upxi}}}_{4}$	${{\boldsymbol{\uplambda}}}$	${{\boldsymbol{\upbeta}}}$	${\mathbf{R}}_{{\mathbf{c}}}$(Ω)	Best SSE (V^2^)
WOA	− 0.8532452	0.00321545	8.1556	− 0.000172233	23.9	0.013656639	0.000107379	0.83464137
AOA	− 1.17080145	0.00350529	3.66	− 0.000171451	24	0.0136	0.0001	0.884768219
GWO	− 0.99474929	0.00325367	5.57436	− 0.000166649	24	0.016069242	0.000100086	0.814099689
MVO	− 1.05459991	0.00394192	9.25241	− 0.000166411	24	0.016106354	0.000100481	0.814352894
EDO	− 0.89959206	0.00346504	9.05769	− 0.000167251	24	0.015885386	0.0001	0.813911722
FTO	− 0.96740502	0.00347621	7.72427	− 0.000167252	24	0.015883515	0.0001	0.81391171
EFTO	− 1.04489966	0.00316821	3.90746	− 0.000167251	24	0.015884374	0.0001	0.813911703

**Figure 18:**
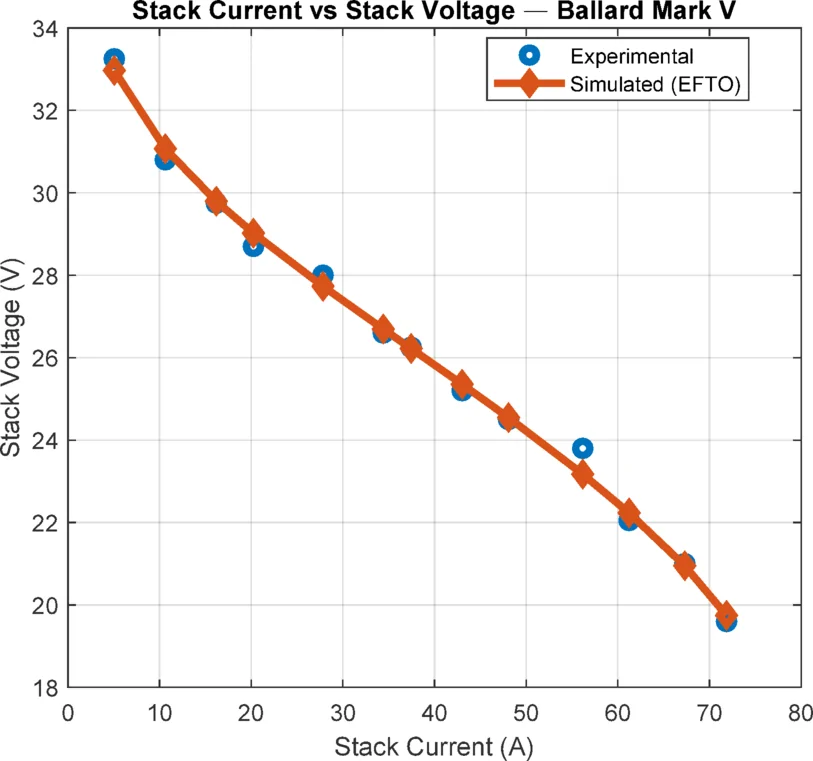
Ballard Mark V: experimental vs EFTO-simulated I-V polarization curve.

**Figure 19:**
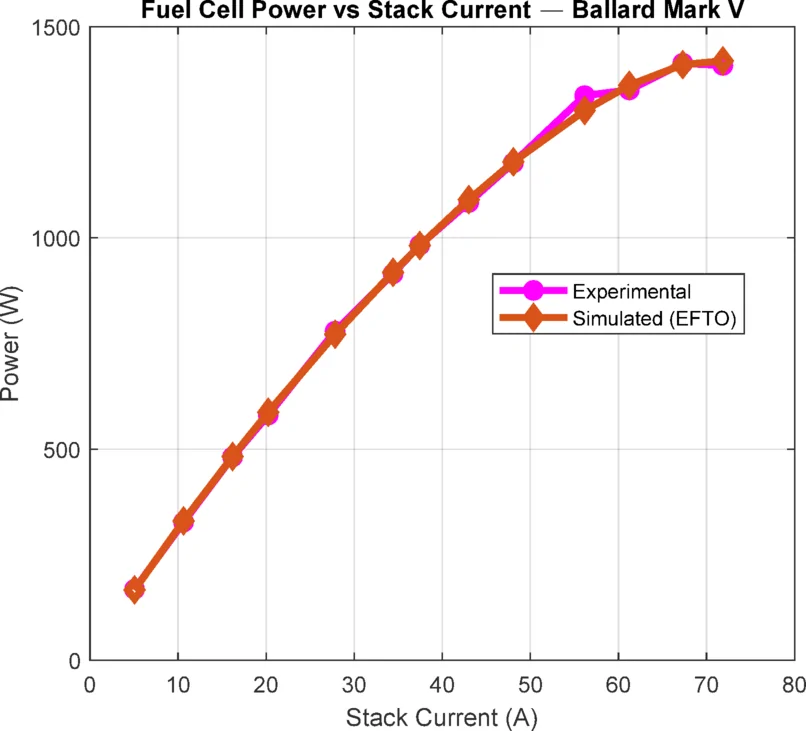
Ballard Mark V: experimental vs EFTO-simulated I-P polarization curve.

The I-V curve (Fig. 18) reproduces all 13 experimental points with max error 2.634% at 56.166 A, mean error 0.742%, and RMSE = 0.2502 V. Predicted peak power: 1,418.89 W at 71.852 A (Fig. 19). Analysis of voltage decomposition confirms E_Nernst_ = 41.681 V (the lowest across the three stacks due to the 35-cell configuration), V_act_ dominating at 8.355–13.682 V (52.2–69.2% of total drop) and partway through an intermediate regime for V_con_ (0.038–1.630 V) between NedStack PS6 and SR-12^9,14,23,62^.

**Figure 20:**
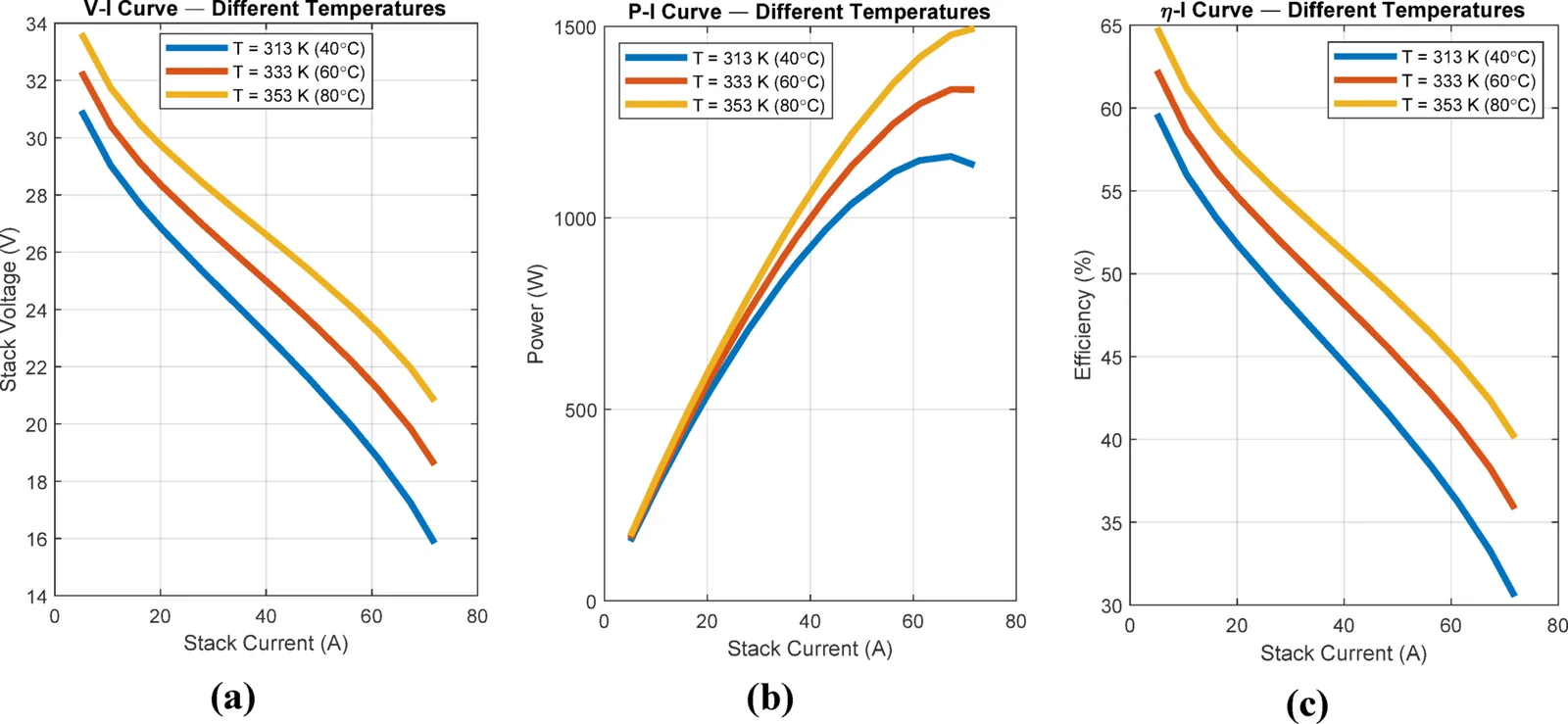
Ballard Mark V at three temperatures (313 K, 333 K, 353 K): (**a**) I-V curves; (**b**) I-P curves; (**c**) I-η efficiency curves.

**Figure 21:**
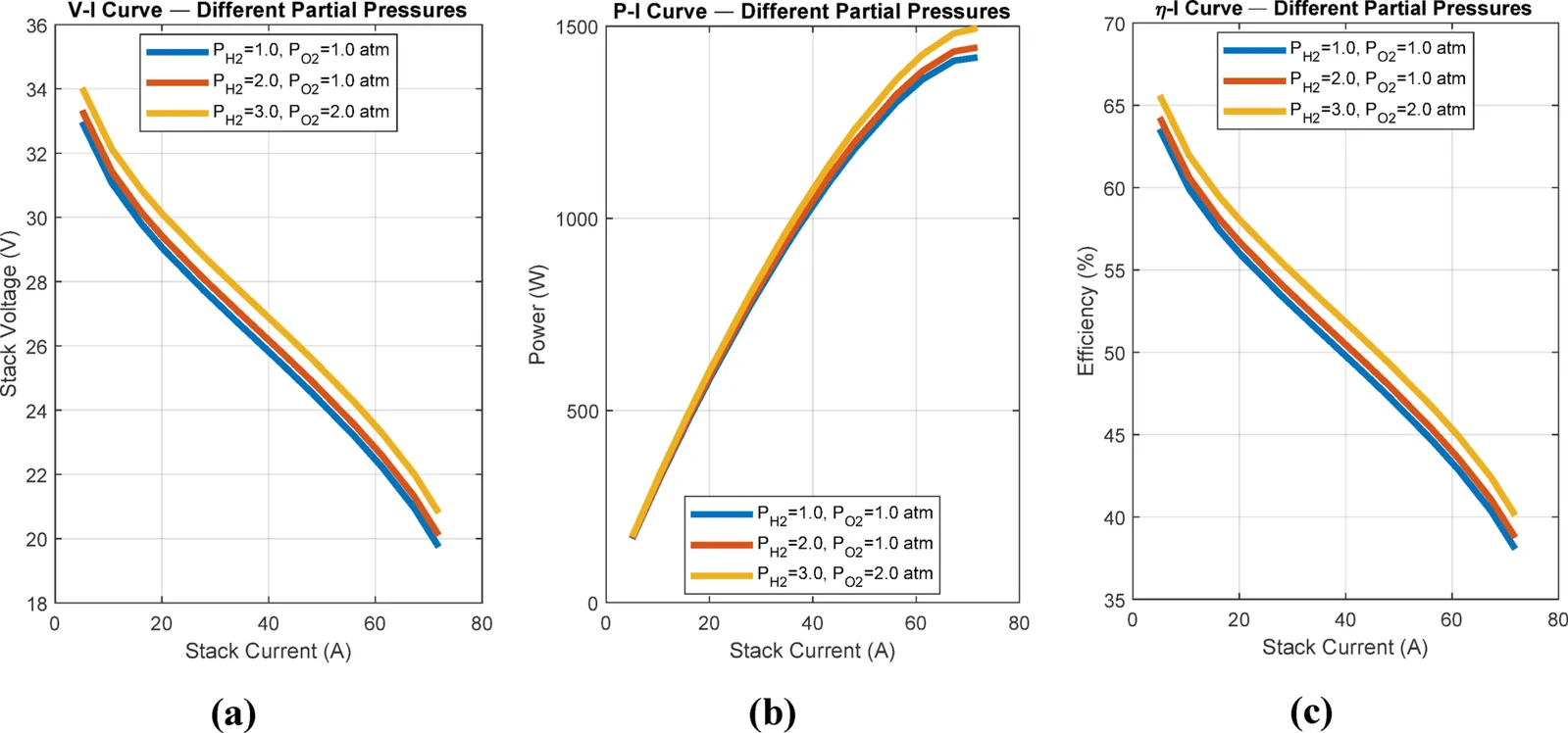
Ballard Mark V at three partial pressure combinations (P_H₂_/P_O₂_ = 1.0/1.0, 2.0/1.0, 3.0/2.0 atm): (**a**) I-V curves; (**b**) I-η efficiency curves.

Temperature sensitivity (Fig. 20) at 313 K, 333 K, and 353 K peak power is raised from 1,160.87 W to 1,335.51 W (+ 15.0%) and 1,495.05 W (+ 28.8%), the greatest temperature-driven increase of the three stacks. Fuel cell efficiency of 30.531% at 71.852 A improved to 40.112% (Tables 14, 15). Pressure sensitivity (Fig. 21) demonstrates OCV increasing + 4.49% and terminal voltage at 71.852 A improving + 7.51% at P_H₂_/P_O₂_ = 1.0/1.0, 2.0/1.0, and 3.0/2.0 atm from elevating pressure^59^.

**Table 14:** Statistical analysis, Friedman rank, and Wilcoxon test—Ballard Mark V (30 runs).

Metric	WOA	AOA	GWO	MVO	EDO	FTO	EFTO
Min SSE (V^2^)	0.834641	0.884768	0.814100	0.814353	0.813912	0.813912	0.813912
Max SSE (V^2^)	2.296151	2.758007	0.851538	1.019597	0.813944	0.813931	0.813912
Mean SSE (V^2^)	1.476288	1.305171	0.823413	0.883862	0.813916	0.813915	0.813912
Std (V^2^)	0.400277	0.378240	0.011536	0.066528	6.68 × 10⁻⁶	5.38 × 10⁻⁶	1.289 × 10⁻^15^
Friedman Rank	7	6	4	5	3	2	1
R⁺ (Wilcoxon)	465	465	465	465	465	465	N/A
*p*-value	1.73 × 10⁻⁶	1.73 × 10⁻⁶	1.73 × 10⁻⁶	1.73 × 10⁻⁶	1.73 × 10⁻⁶	1.73 × 10⁻⁶	N/A
Result vs EFTO	+	+	+	+	+	+	Ref

**Table 15:** Sensitivity analysis: SSE response to ± 5% parameter perturbation, Ballard Mark V.

Parameter	Optimal Value	+ 5% SSE (V^2^)	ΔSSE% (+ 5%)	− 5% SSE (V^2^)	ΔSSE% (− 5%)	Rank
ξ₁	− 1.044900	44.2810	5.34 × 10^3^	44.2826	5.34 × 10^3^	2
ξ₂	0.003168	47.8297	5.78 × 10^3^	47.8281	5.78 × 10^3^	1
ξ₃	3.907 × 10⁻^5^	2.2133	171.93	2.2135	171.96	4
λ	24.000	1.2878	58.23	1.5124	85.82	5
ξ₄	− 1.673 × 10⁻^4^	2.4265	198.13	2.4268	198.16	3
Rc	1.000 × 10⁻^4^	0.8163	0.29	0.8130	− 0.11	7
β	0.015884	0.8299	1.97	0.8300	1.97	6

The result of the Wilcoxon signed-rank test gives R⁺ = 465, *p* = 1.73 × 10⁻⁶ with all pairwise comparisons, confirming that EFTO performs a consistently lower SSE across all 30 independent runs. The best SSE values obtained by EFTO and FTO differ only in the eighth digit: 0.813911703 V^2^ and 0.813911710 V^2^ respectively, indicating differences smaller than any physically meaningful threshold, but solution reproducibility itself remains a definitive criterion that sets apart EFTO and FTO as two distinct methods. A standard deviation of 1.289 × 10⁻^15^ V^2^ is obtained for EFTO, as compared to 5.382 × 10⁻⁶ V^2^ for FTO, an improvement of nearly a factor of nine orders of magnitude. The same globally stable solution is guaranteed to be converged on if initialized at random positions, meaning the EFTO algorithm is extremely robust and suitable for integration in any engineering applications deploying optimization-based parameter identification.

Parameter ξ₂ (rank 1, ΔSSE = 5,777%) becomes the most sensitive parameter and leaves behind ξ₁ (rank 2, ΔSSE = 5,341%), which is the only other sensitive factor that consistently completes the top-two ranking of NedStack PS6 and AVISTA SR-12. With λ = 24, confirms full membrane saturation at 343 K, and the very large value for ξ₄ mirrors strong temperature-current coupling consistent with enhanced catalyst deactivation as a function of increasing temperature. The dominant sensitivity of ξ₁ and ξ₂ confirms activation overpotential as the primary loss mechanism (V_act_ = 52.2–69.2% of total voltage drop), while Rc (rank 7, ΔSSE = 0.29%) and β (rank 6, ΔSSE = 1.97%) are insensitive. EFTO’s Std = 1.289 × 10⁻^15^ V^2^ guarantees physically consistent parameter recovery across all 30 independent runs.

### Strengths and limitations of EFTO

The results reveal three consistent strengths. First, machine-precision reproducibility (Std < 10⁻^14^ V^2^) across all 30 runs on all three PEMFC stacks confirms that the co-designed architecture reliably eliminates run-to-run variance. Second, the ablation study confirms synergistic mechanism interaction: disabling all mechanisms collectively yields + 3032.8% SSE degradation, substantially exceeding the sum of individual removals. Third, EFTO achieves the lowest average runtime among all four state-of-the-art algorithms at both D = 50 (8.653 s) and D = 100 (12.424 s). Three limitations are also identified. First, EFTO is outperformed by one competitor at D = 50 (*p* = 0.023), indicating the covariance-adaptive sampling branch may underperform specialised solvers on certain multimodal function classes. Second, M5 and M7 are deactivated at D > 30, restricting the contribution of M5 and M7 to low-dimensional problems only. Third, the study is limited to the Amphlett steady-state model with seven parameters; performance on dynamic models or alternative fuel cell formulations remains unevaluated.

## Conclusion

This paper has presented the Enhanced Fourier Transform Optimizer (EFTO), addressing five structural deficiencies of the baseline FTO through eight synergistic mechanisms. Five contributions are made: (i) a dual-branch hybrid architecture combining success-history adaptive differential evolution with covariance-adaptive population sampling; (ii) a dimension-aware heuristic switching strategy; (iii) an online adaptive branch probability mechanism; (iv) comprehensive CEC 2017 benchmark validation with component-wise ablation; and (v) physically validated PEMFC parameters identification across three commercial stacks. Ablation confirms co-designed synergy: removing all mechanisms yields + 3032.8% SSE degradation (*p* = 2.30 × 10⁻^2^⁶), CAPS removal alone causes + 98.7% (*p* = 1.09 × 10⁻^24^), and elite-guided mutation removal causes + 54.3% (*p* = 3.86 × 10⁻^1^⁰).

In CEC 2017 benchmark suite (D = 50, 28 functions, 30 runs), the best overall performance can be obtained by adopting EFTO which achieves Friedman rank 1 (average rank = 1.607, *p* ≤ 1.17 × 10⁻^3^). On D = 50 (8.653 s) and D = 100 (12.424 s), EFTO is statistically tied with two of the three SOTA optimizers considered (*p* > 0.05) while recording the lowest runtime. The only statistically superior result at D = 50 (*p* = 0.023) is for a competitor that realizes the benefit of EFTO purposefully turning off M5 and M7 in high-dimensional problems, effectively exposing some clearly defined direction to improve upon next.

All three stacks show machine-precision reproducibility (Std < 10⁻^14^ V^2^) for PEMFC parameters identification with corresponding SSEs of 2.0656 V^2^ (NedStack PS6), 1.0564 V^2^ (AVISTA SR-12), and 0.8139 V^2^ (Ballard Mark V). The statistical significance of the machine-precision reproducibility result is confirmed by the Wilcoxon signed-rank test (R⁺ = 465, *p* = 1.73 × 10⁻⁶). The machine-precision reproducibility level offers a practical engineering guarantee: while competing algorithms with standard deviations between 10⁻^3^ and 10⁻⁶ V^2^ may yield inconsistent parameter estimates, EFTO converges to the same globally consistent solution irrespective of initialization. In addition, stack-specific sensitivity analyses confirm the physical validity of the identified parameters beyond fitting performance alone.

Four limitations motivate future work: (i) restriction to the Amphlett steady-state model excludes dynamic and degradation behaviour; (ii) evaluation on three stacks at fixed conditions limits generalisation; (iii) batch identification excludes real-time online adaptation; and (iv) direct comparison with recently proposed algorithms^29,30^ was not conducted due to post-submission publication. Future work will address the four identified limitations through extension to constrained and multi-objective PEMFC formulations, real-time parameter adaptation, solid oxide fuel cell models, and extended high-dimensional benchmarking including^29,30^.

## Supplementary Information

Below is the link to the electronic supplementary material.

Supplementary Material 1.

## Data Availability

The datasets used and/or analyzed during the current study are available from the corresponding author on reasonable request.
