# Supplementary material for: Parameter estimation of proton exchange membrane fuel cells using enhanced Fourier transform optimizer with adaptive learning mechanisms
Source: Sci Rep. 2026 Jul 8;16:26519. doi: 10.1038/s41598-026-60492-z (PMC13503772; doi:10.1038/s41598-026-60492-z)
Supplement: Supplementary file 1 [file 41598_2026_60492_MOESM1_ESM.docx]

Appendix

Table A1.Friedman rank summary and Wilcoxon signed-rank test results on CEC 2017 (D = 100).

| **Metric** | **EFTO** | **LSHADE** | **CMA-ES** | **jSO** |
| --- | --- | --- | --- | --- |
| Avg. Friedman Rank | 2.2857 | 2.7857 | 1.8214 | 3.1071 |
| Final Rank | 2 | 3 | 1 | 4 |
| Wilcoxon p-value | — | 1.11E-01 | 4.25E-01 | 3.51E-01 |
| Result vs EFTO | Reference | No significant difference | No significant difference | No significant difference |
| Run Time in Sec | 12.424 | 13.164 | 15.482 | 13.545 |
